# Blood samples collected under anesthesia can be used as a source of non-diseased controls for immune-based assays

**DOI:** 10.3389/fimmu.2025.1618080

**Published:** 2025-07-24

**Authors:** Clara Domingo-Vila, Evangelia D. Williams, Megan E. Smithmyer, Basilin Benson, Lauric A. Ferrat, Sefina Arif, Michelle Hudson, Becky Dobbs, Matthew B. Johnson, Iain Yardley, Cate Speake, Richard A. Oram, Timothy I. M. Tree, Benjamin J. Blaise, EXE-T1D consortium

**Affiliations:** ^1^ School of Immunology and Microbial Sciences, King’s College London, London, United Kingdom; ^2^ Center for Interventional Immunology, Benaroya Research Institute, Seattle, WA, United States; ^3^ Center for Systems Immunology, Benaroya Research Institute, Seattle, WA, United States; ^4^ Department of Clinical and Biomedical Sciences, University of Exeter Medical School, Exeter, United Kingdom; ^5^ Department of Genetic Medicine and Development, University of Geneva, Geneva, Switzerland; ^6^ Guy’s and St Thomas’ NHS Foundation Trust, Evelina London Children’s Hospital, Department of Paediatric Surgery, London, United Kingdom; ^7^ Guy’s and St Thomas’ NHS Foundation Trust, Evelina London Children’s Hospital, Paediatric Anaesthetic Department, London, United Kingdom; ^8^ King’s College London, Centre for the Developing Brain, St Thomas’ Hospital, London, United Kingdom

**Keywords:** anaesthesia, blood, children, immune cells, controls

## Abstract

Recruiting very young, healthy children to serve as age-matched controls in research presents substantial ethical and practical challenges. One potential approach to address this issue is to recruit healthy children who are referred for elective procedures under general anesthesia. As infants are typically anesthetized using volatile anesthetics before cannula insertion for additional drug administration, blood samples become readily accessible after the onset of drug-induced coma. However, since prolonged exposure to inhaled anesthetic agents is known to have immune-modulating effects that could affect their suitability as experimental controls, we aimed to investigate whether immune changes are also present in samples collected immediately after gas induction in children undergoing elective dental procedures. The composition and transcriptional profile of whole blood immune cells were assessed using multiparameter flow cytometry and bulk RNA-sequencing, respectively. Cryopreserved PBMCs were used to study changes in the phenotype of polyclonally activated CD4^+^ T cells by single-cell RNA sequencing using the 10x Genomics (Pleasanton, CA, USA) platform. We report that inhaled anesthetic induction with a combination of nitrous oxide and sevoflurane has minimal effect on immune system composition and transcriptional profiles, and does not alter the phenotype of CD4^+^ T cells activated with staphylococcal enterotoxin B (SEB). However, we observed increased absolute cell counts in specific leucocyte populations. We conclude that blood samples collected during elective procedures under general anesthesia may represent a valuable opportunity for recruiting healthy children for research studies, depending on the intended assays.

## 1 Introduction

Alterations in the immune composition and/or function may contribute to the development of various disorders, including those affecting very young children (1, 2). However, accurately identifying such changes depends on the selection of an appropriate comparator population, with careful control for genetic, environmental, and age-related factors. Studies examining the immune system across different age groups are particularly challenging due to the profound influence of aging on the immune system. Continuous interactions with the microbial environment shape the immune system throughout an individual’s lifetime (3, 4). Age alters the frequency and function of blood immune cells, contributing to a decline in immune responses over time (5). Strong positive and negative associations between the frequency of various immune cell populations and age have been identified (5, 6). The most prominent examples are observed within the adaptive immune system, where age-associated changes in circulating lymphocytes have been widely reported and are considered a hallmark of immune system aging (7). These changes are particularly pronounced during early childhood, when the frequencies of different immune cell types change dynamically as the immune system matures in response to infections, vaccinations, and environmental exposures.

Obtaining appropriately age-matched controls is particularly challenging in pediatric research due to a combination of ethical and practical considerations. One potential solution is the recruitment of healthy children referred for elective procedures performed under general anesthesia. The ability to collect a blood sample while the child is asleep substantially reduces the difficulties associated with venepuncture, as well as the stress experienced by patients, parents, and healthcare professionals. Moreover, intravenous (IV) cannulation is a mandatory step in any anesthetic procedure, enabling the administration of fluids and medications or the rapid delivery of emergency drugs in the event of unexpected complications. Pediatric anesthetists are therefore uniquely positioned to collect blood samples from children across all age groups.

General anesthetic agents can be administered intravenously or via inhalation using a face mask (8). In pediatric settings, inhalation is often used to avoid the discomfort and difficulties associated with cannulation in babies and toddlers, which are primarily due to increased fatty tissue, fragile veins, and patient movement. Inhalational agents, in particular sevoflurane, are the most commonly used method for inducing general anesthesia in neonates and infants. They allow for a smooth induction without the need for initial cannulation, resulting in rapid loss of consciousness. After this initial phase, a cannula is usually inserted before airway management, while the child is unconscious, immobile, and free of pain. Cannulation is facilitated by the vasodilatory properties of hypnotic agents, although ultrasound guidance may occasionally be required.

Several studies conducted to date have indicated that exposure to volatile anesthetics (VAs) can influence immune system measurements, including changes in cell frequency, count, function, and/or cytokine secretion (8–11). However, these studies typically compare samples taken pre- and postoperatively, thereby capturing a combination of factors, including the effects of prolonged exposure to VAs and operative-associated stress. In this study, we collected blood samples from children undergoing elective dental procedures both before and shortly after inhaled anesthetic induction with nitrous oxide and sevoflurane. We investigated whether aesthesia induces immediate transcriptional changes or alterations in immune cell frequency, number, and function, to evaluate the suitability of these samples as nondiseased pediatric controls in immunological research.

## 2 Materials and methods

### 2.1 Patient recruitment and blood sampling

This study recruited a total of 10 individuals between 3 and 6 years of age who were referred for elective dental procedures under general anesthesia (**Table 1**). Families were approached before the day of the procedure to discuss the study protocol and explain how it would deviate from the standard anesthetic protocol. On the day of the procedure, prilocaine cream was applied to both antecubital fossae for at least 15 min to facilitate the insertion of a 22-G cannula into the cephalic or median cubital veins. Analgesic cryogenic ethyl chloride spray was applied after skin preparation, and ultrasound guidance was used if the vein could not be visualized or felt. The first blood sample was collected before the administration of any anesthetic gases (pre-SEVO). At this point, parents were asked to confirm consent for inhalational induction. A mixture of nitrous oxide and oxygen was then administered, with the concentration of sevoflurane gradually increased to 8%. Once the child was asleep, nitrous oxide was turned off, and a second blood sample was collected via the cannula (post-SEVO). Hemodynamic and oxygen stability were maintained throughout this step. Sevoflurane was then reduced to 3%, and additional anesthetic drugs were administered to facilitate airway management before proceeding with the planned dental procedures. This study was carried out with the approval of the UK National Research Ethics Service (EXE-T1D, REC ref 17/EM/0255).

**Table 1:** Demographic data of individuals recruited into the study.

Individual	Age (years, months)	Sex	Ethnicity
P1	5, 8	F	Asian
P2	5, 2	F	White
P3	4, 0	F	Asian
P4	5, 2	M	Asian
P5	3, 10	F	Mixed
P6	6, 9	F	Other
P7	6, 1	F	Mixed
P8	3, 5	M	Black
P9	5, 6	F	Asian
P10	5, 2	F	Mixed

### 2.2 PBMC isolation and cryopreservation

Fresh heparinized blood was diluted 1:1 with complete medium (RPMI-1640 GlutaMAX supplemented with penicillin–streptomycin [Thermo Fisher Scientific, Waltham, MA, USA]). The diluted blood was carefully overlaid into Leucosep tubes containing Lymphoprep (Axis-Shield Diagnostics Ltd, Dundalk, Ireland) and centrifuged at 1,000 × *g* for 15 min at room temperature (RT). The plasma layer was carefully removed and discarded, and the Peripheral Blood Mononuclear Cells (PBMC) layer was washed twice with complete media at 300 and 200 × *g* for 10 min each. After determining the total cell number, Peripheral Blood Mononuclear Cells (PBMC) layer were centrifuged at 400 × *g* for 5 min, resuspended in CryoStor freezing medium (Sigma-Aldrich, St. Louis, MO, US), and transferred into cryogenic vials (Corning, Corning, NY, USA). Vials were placed in a controlled-rate freezing vessel (CoolCell, Biocision, San Rafael, CA, USA) at – 80°C overnight before being transferred to temperature-monitored LN2 tanks for long-term storage.

### 2.3 Flow cytometric analysis of fresh whole blood

Leukocyte populations were characterized from fresh whole blood (collected in 2.7 mL Ethylenediaminetetraacetic Acid (EDTA) tubes) using five multiparameter flow cytometry panels previously validated and assessed for technical reproducibility across multiple laboratories (12, 13). In brief, surface marker staining (**Supplementary Tables S1**-**S4**) was performed on 100–200 μL of well-mixed fresh whole blood for 45 min at RT, followed by red blood cell lysis for 8 min at RT (10× BD Fluorescence-Activated Cell Sorting (FACS) lysing solution diluted in Double Distilled Water (ddH_2_O), BD Biosciences, USA). Staining for the lineage flow cytometry panel was performed using BD Trucount tube (BD Biosciences, Franklin Lakes, NJ, USA), allowing for the calculation of absolute cell numbers in addition to cell frequencies. The lineage tube was vortexed to ensure homogenization and kept on ice until acquisition (lyse, no wash). All other staining panel tubes were centrifuged at 500 × *g* for 5 min, washed in 2 mL of FACS buffer (1 × phosphate-buffered saline [PBS; Invitrogen, USA] containing 0.2% BSA [Sigma-Aldrich, USA] and 2 mM EDTA [Sigma-Aldrich, USA]) by centrifugation at 500 × *g* for 5 min, resuspended in 200 μL of FACS buffer, and kept on ice until acquisition. Intracellular marker staining (**Supplementary Table 5**) of 100 μL of fresh whole blood consisted of two 15-min incubations at RT: first with CD45RA-BV785, followed by 10 μL of fixative reagent (buffer 1 from PerFix-nc kit, Beckman Coulter, Brea, CA, USA), respectively. Permeabilizing reagent (buffer 2 from PerFix-nc kit, Beckman Coulter, USA) was added to the intracellular master mix of fluorescently labeled antibodies prepared in advance, and cells were stained intracellularly for 60 min at RT. After incubation, 3 mL of plain 1 × PBS (Invitrogen, USA) was added for 5 min, followed by centrifugation at 500 × *g* for 5 min. Cells were washed in 3 mL of 1 × R3 reagent (10 × buffer 3 diluted in ddH_2_O from PerFix-nc kit, Beckman Coulter, USA), resuspended in 200 μL of 1 × R3 reagent, and kept on ice until acquisition.

### 2.4 Bulk RNA-sequencing analysis

RNA-seq was performed on venous whole blood samples collected from participants pre- and post-SEVO. Samples were stabilized using the Tempus Spin RNA Isolation Kit (Applied Biosystems, Waltham, MA, USA), as previously described (14). Base calls were processed to FASTQ files on BaseSpace (Illumina, San Diego, CA, USA), and quality trimming was applied to remove low-confidence base calls from the ends of reads. FASTQ files were aligned to the University of California Santa Cruz (UCSC) Human genome assembly version 38.91 using STAR v.2.4.2a. Duplicate filtering, quality control, and metrics analysis were performed using tools from the Picard 1.134 software suite (1). For quality control, samples were required to have > 1 million human-aligned mapped reads and a median coefficient of variation (CV) coverage < 0.6. Genomic variants called from the RNA-seq reads were used to verify RNA-seq sample identity using kinship comparisons (14). Sex-chromosome-associated gene expression was used to confirm that participant sex matched metadata annotations. Genes were filtered to include those with a trimmed mean of *M*-values (TMM) normalization count of at least 0.8 in at least two samples and further restricted to protein-coding genes. The final dataset included 19 samples comprising 14,590 genes.

### 2.5 Single-cell RNA-sequencing analysis

Cryopreserved PBMCs from five individuals (pre- and post-SEVO paired time points) were cultured in RPMI-1640 GlutaMAX medium (Thermo Fisher Scientific, USA) supplemented with penicillin–streptomycin (Thermo Fisher Scientific, USA), Fungizone/amphotericin B (Thermo Fisher Scientific, USA), 10% fetal bovine serum (FBS, Thermo Fisher Scientific, USA), and InVivoMAb antihuman CD40 (Bio X Cell, Lebanon, NH, USA). Cells were either left unstimulated or stimulated overnight at 37°C with 50 ng/mL superantigen enterotoxin B (SEB; Sigma-Aldrich, USA). Following cell stimulation, cells were harvested and stained at 4°C for 30 min with fluorescently labeled antibodies, TotalSeq-C reagents (**Supplementary Table S6**), and a combination of TotalSeq-Hashtag antibodies from BioLegend and Abcam. After staining, cells were washed twice with FACS buffer (1 × PBS (BioLegend, San Diego, CA, USA) containing 1% human AB serum (Sigma-Aldrich, US) and 2 mM EDTA (Sigma-Aldrich, USA]) by centrifugation at 400 × *g* for 5 min, followed by staining with live/dead (L/D) marker (7-AAD viability staining solution, BioLegend, USA). Samples were sorted on a BD FACSAria II flow cytometer (gating strategy shown in **Supplementary Figure S6**) and processed using the 10 × Chromium Next GEM Single Cell 5′ sequencing platform with Feature Barcode technology (10× Genomics, USA). Libraries were sequenced on a NextSeq 2000 sequencing system (Illumina, USA).

### 2.6 Statistical analysis

Principal component analyses (PCA) were performed on centered and scaled frequency data and log2-transformed expression data using the prcomp function from the built-in stats package in RStudio (version 4.2.2). Agglomerative hierarchical clustering analysis based on Euclidean distances was performed using the pheatmap package (version 1.0.12) in RStudio. Clustering was performed between immune parameters/samples on centered and scaled frequency data using Ward’s method, and between the 100 most variable genes/samples on centered and scaled log2-transformed expression data using the complete linkage method. The 100 most variable genes were identified by calculating the variance across all libraries using base R functions for each gene. Genes related to sex were excluded from this list. Comparison of immune cell frequencies between pre- and post-SEVO samples was performed using multiple paired *t*-tests with or without correction for multiple comparisons using the Bonferroni–Dunn method, in GraphPad Prism version 9.0.0. To assess the effect of gas induction relative to interindividual variation, a linear mixed model was applied. The ratio of variance explained by inhaled anesthetic induction versus variance explained by interindividual differences was calculated (15). Volcano plots were generated using the R package ggplot2 (version 3.4.4) on differentially expressed gene results from limma (16). Single-cell RNA-sequencing FASTQ files were generated using Cell Ranger (v7.2.0) mkfastq pipeline from the bcl files. Samples were then processed through the Cell Ranger multipipeline to generate feature-barcode matrices. Dehashing and doublet marking were performed using the Seurat package. Data were imported into Seurat (v4.2.0) to generate a category file compatible with the cloupe file format for downstream analysis using the Loupe Browser (10× Genomics, USA). Differences in the proportion of cells within each of the four clusters identified by graph-based clustering were assessed by Chi-squared analysis. A *p-*value of < 0.05 was considered statistically significant. Comparisons without labels indicate nonsignificant differences.

## 3 Results

### 3.1 Sevoflurane administration does not alter immune cell subset frequencies

**Figure 1:**
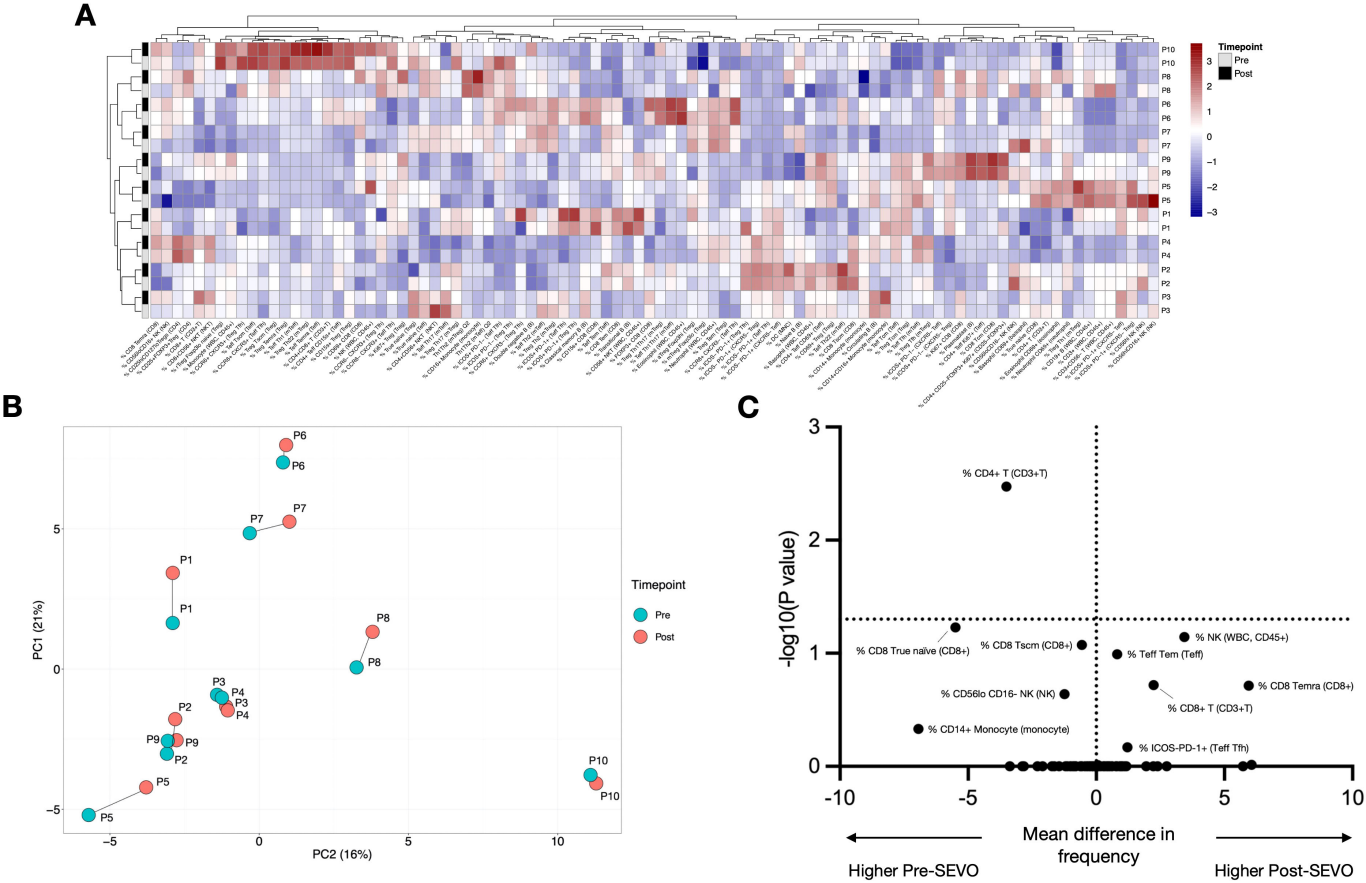
Minimal changes in immune system composition after sevoflurane induction. **(A)** Agglomerative hierarchical clustering analysis of changes in cell population frequency is represented as a heatmap. Patient IDs are shown vertically on the right-hand side of the heatmap (*y*-axis), and cell populations are shown horizontally below the heatmap (*x*-axis). Samples before induction are shown in grey, and samples after induction are shown in black. **(B)** Principal component analysis (PCA) of blood samples from 10 individuals (P1–P10) collected before and after inhaled anesthetic induction, indicated as “pre” and “post”, respectively. The two principal components (PCs) are shown. Samples from the same individual are joined by a line. **(C)** Multiple paired *t*-tests were performed with correction for multiple comparisons using the Bonferroni–Dunn method. The threshold for a significant mean difference in cell frequency before and after inhaled anesthetic induction is represented by a dotted line at *y* = 1.3 (alpha = 0.05).

Flow cytometric staining was conducted using five panels of monoclonal antibodies to measure the frequency of granulocyte, T cell, B cell, dendritic cell (DC), monocyte, and natural killer (NK) cell subsets, and assess T-cell activation and proliferation (example Boolean gating schemes are shown in **Supplementary Figures S1**-**S5**). A total of 100 leukocyte subpopulations were enumerated. To evaluate the effect of anesthetic induction, unbiased hierarchical clustering analysis was conducted on standardized population frequencies from pre- and post-SEVO samples, with the resulting clustered heatmap shown in **Figure 1A**. In all cases, paired blood samples from each individual clustered together, indicating that samples collected after inhaled anesthetic induction were highly representative of those collected prior to induction. These findings were supported by principal component analysis (PCA), which showed that paired samples were located in close proximity on the PCA plot (**Figure 1B**).

In addition to assessing whether sevoflurane affected the overall composition of the immune system, we evaluated its impact on individual immune cell populations using multiple paired *t*-tests (with or without correction for multiple comparisons). After applying the Bonferroni–Dunn correction, the frequency of a single population (CD4^+^ T cells) was significantly altered following induction (**Figure 1C**). Without correction for multiple comparisons, this number increased to 22/100 immune cell subsets, including NK cells, monocyte subpopulations, and CD4^+^ and CD8^+^ T cells (**Supplementary Table S7**; **Supplementary Figure S7**). To contextualize these differences relative to interindividual variation, we applied a linear mixed model, which revealed that for 92/100 cell populations, the variance explained by gas induction was at least 10 times smaller than the variance explained by individual differences (**Supplementary Table S8**). Four cell populations (% CD56hi NK, % CD56loCD16^+^ NK, % CD69^+^ CD8, and % Total NK) exhibited an effect where gas induction accounted for more than 30% of the variance. Overall, these results suggest that inhaled anesthetic induction has a minimal effect on a limited number of cell subsets, especially when compared to the variation observed between individuals.

When assessing absolute cell counts using the BD Trucount tube (allowing calculation of cells/µL of blood), we observed a small but significant increase in total leukocyte number following sevoflurane induction (mean fold change ± SD: 1.26 ± 0.43). This significant increase was observed across all 17 subpopulations assessed, with the greatest effects seen in NK cells, monocytes, including their subsets (monocyte mean fold change: 3.03 ± 1.68; NK cell mean fold change: 1.95 ± 2.05). These changes also showed the highest interindividual variability (**Supplementary Figures S8, S9**).

### 3.2 PBMC yields are increased after sevoflurane administration

Consistent with the increased fold change in cell number observed by flow cytometry, we also detected a significant increase in PBMC yield following sevoflurane administration (mean ± SD fold change: 1.53 ± 0.71) (**Supplementary Figure S10**).

### 3.3 The transcriptional profile of whole blood immune cells remains stable after sevoflurane induction

**Figure 2:**
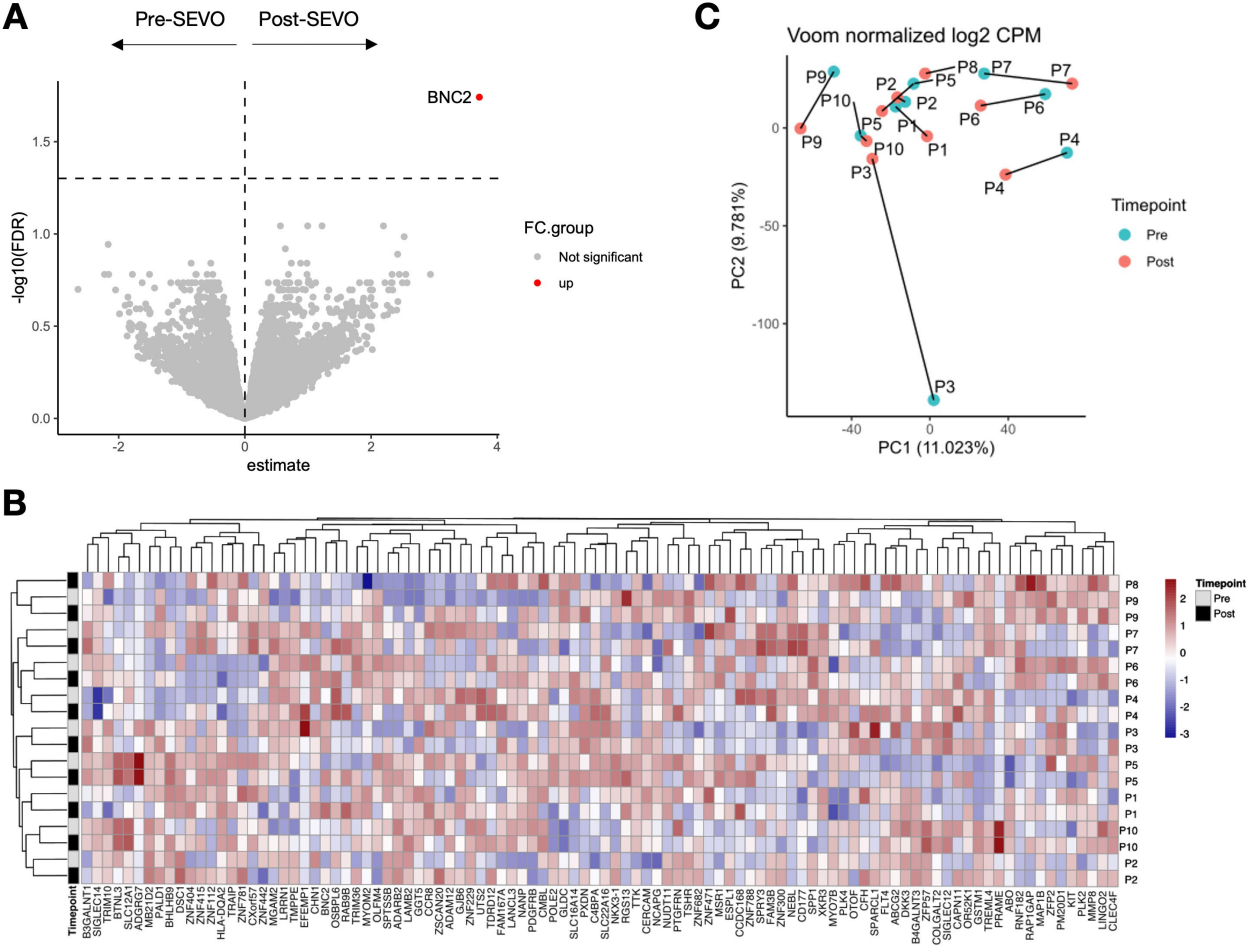
Whole blood RNA-sequencing analysis shows minor changes in gene expression after sevoflurane administration. **(A)** Volcano plot showing log2 fold change (*x*-axis) and the logarithm of the corrected *p*-value (*y*-axis) for 14,590 protein-coding genes identified in 19 samples. **(B)** Agglomerative hierarchical clustering analysis of changes in cell population frequency is represented as a heatmap. Patient IDs are shown vertically on the right-hand side of the heatmap (*y*-axis), and the 100 most variable genes are shown horizontally below the heatmap (*x*-axis). Samples before induction are shown in grey, and samples after induction are shown in black. **(C)** PCA of blood samples from 10 individuals (P1–P10) collected before and after inhaled anesthetic induction, indicated as “pre” and “post”, respectively. The two principal components (PCs) are shown. Samples from the same individual are connected by a line.

Whole blood transcriptional profiling identified a total of 14,590 protein-coding genes, of which only one—BNC2—was significantly altered following sevoflurane induction (fold change = 3.71, FDR = 0.02) (**Figure 2A**). Unbiased hierarchical clustering analysis of the 100 most variable genes was performed to investigate gene expression differences between pre- and post-SEVO samples, with the resulting clustered heatmap shown in **Figure 2B**. Paired samples from the same individual clustered together, indicating that interindividual variation in gene expression was more pronounced than changes induced by inhaled anesthetic induction. These findings were further supported by PCA, which showed that pre- and post-SEVO samples from each subject were closely clustered on the PCA plot (**Figure 2C**).

### 3.4 Sevoflurane administration does not change the transcriptional profile of activated CD4^+^ T cells at a single-cell resolution

**Figure 3:**
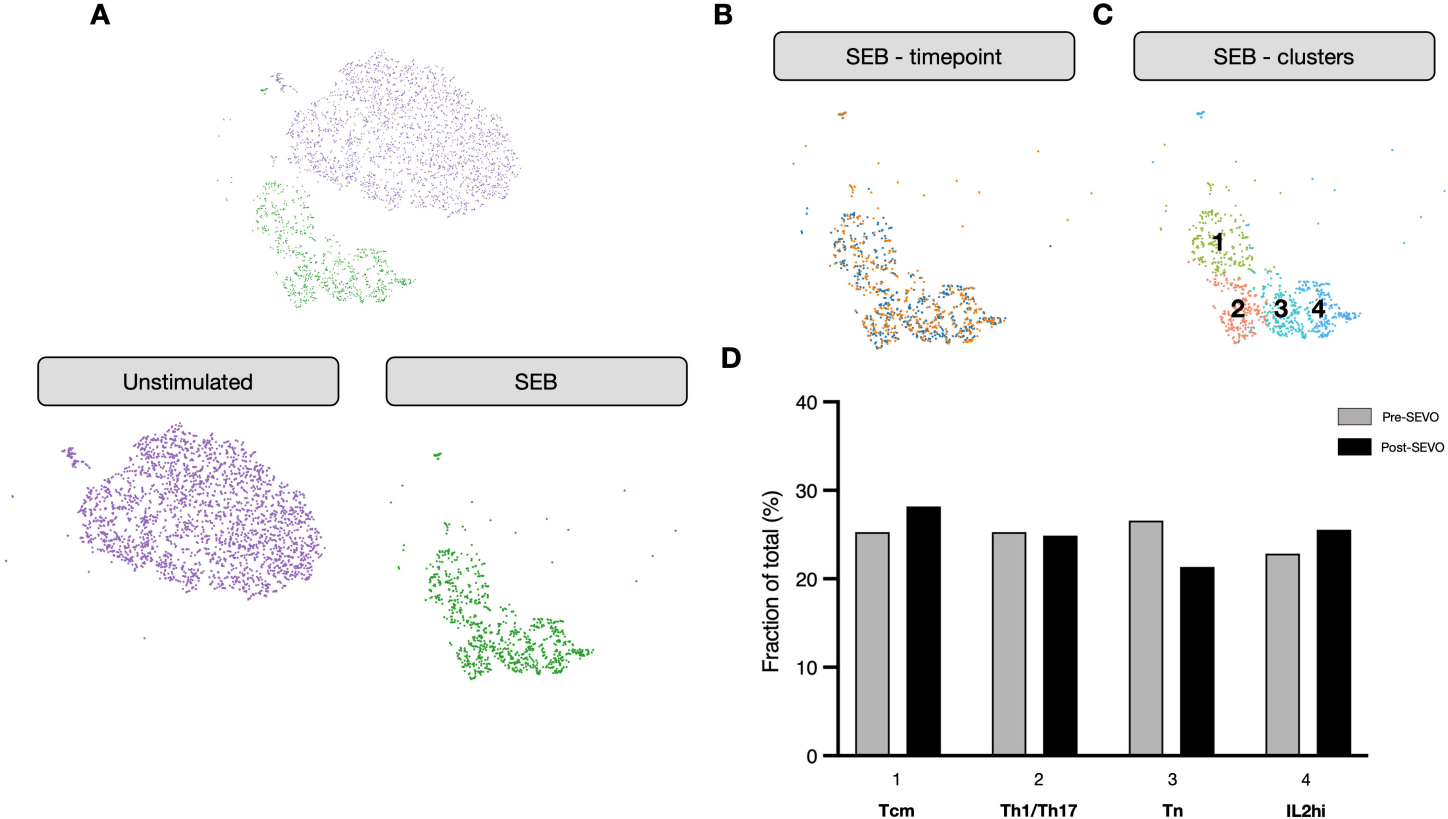
The transcriptional profile of activated CD4^+^ T cells is unaltered after sevoflurane induction. **(A)** Clustering analysis of unstimulated (purple) and SEB-stimulated (green) CD4^+^ T cells from four donors projected onto two dimensions using t-SNE. **(B)** SEB-stimulated CD4^+^ T cells colored by time point (pre-SEVO, blue; post-SEVO, orange). **(C)** Graph-based clustering of SEB-stimulated CD4^+^ T cells. **(D)** Bar chart showing the proportion of cells in each cluster by time point (pre-SEVO, grey; post-SEVO, black). Chi-squared tests performed on all four clusters between pre- and post-SEVO samples showed no significant differences.

To investigate whether sevoflurane affected the functional response of CD4^+^ T cells, we isolated activated T cells using an activation-induced marker (AIM) assay following incubation with the oligoclonal activator SEB. These cells were then profiled using single-cell RNA sequencing (scRNA-seq). As expected, cells from activated and nonactivated culture conditions clustered separately (**Figure 3A**). Analysis by time point revealed no discernible differences in clustering between pre- and post-SEVO samples (**Figure 3B**). To investigate the phenotype of the responding cells, graph-based clustering was performed on SEB-stimulated conditions, which separated the cells into four distinct clusters (**Figure 3C**). Enumeration of the proportion of cells within each cluster revealed no differences between samples collected before and after inhaled anesthetic induction (**Figure 3D**).

## 4 Discussion and conclusions

A major challenge in pediatric studies that assess immune cell population frequency, phenotype, or function in individuals with a pathological condition is the need for a well age-matched, nondisease control cohort. A commonly used approach is the recruitment of first-degree relatives (i.e., siblings), as this is often more feasible for both ethical and practical reasons (e.g., higher chances for consent) (17, 18). However, due to their shared genetic background, siblings may not be the most appropriate independent nondisease control cohort. An alternative approach is the recruitment of children referred to the hospital for elective procedures under general anesthesia (typically hernia repair, cleft surgery, circumcision, or MRI), whose medical conditions do not directly impact immune system composition. Blood samples are regularly drawn under general anesthesia for clinical purposes when a child is scheduled for such procedures. Recognizing that these samples can also be used for research, without any deviation from the standard anesthetic protocol, helps facilitate parental engagement and consent, while avoiding a traumatic experience for the child.

Several research groups have extensively reported on the effects of the volatile inhaled anesthetic sevoflurane on immune cells. The effects include immune suppression (affecting both immune response and cytokine production), inhibition of T-cell proliferation and function (particularly in CD8^+^ T cells), and altered function of various innate immune cell types (such as impaired phagocytic activity of macrophages) (10, 11). However, these findings have been reported either *in vitro* or following prolonged exposure (postoperatively, 24 h, or up to 1 month after surgery) (19, 20), where they are also compounded by the physiological stress of surgery.

Here, we explored the potential use of blood samples collected immediately after inhaled anesthetic induction in children undergoing elective procedures under general anesthesia. Paired samples (obtained before and immediately after anesthetic induction using a combination of nitrous oxide and sevoflurane) revealed minimal changes in the frequency and transcriptional profile of whole blood immune cells, as well as in the phenotype of activated CD4^+^ T cells stimulated with SEB. It is important to note that, before correcting for multiple comparisons, small changes in cell frequency were observed following anesthetic induction, mainly among CD16-expressing cell populations. The concurrently elevated expression levels of the transcription factor basonuclin zinc finger protein 2 (BNC2) suggest rapid activation of an innate immune response to the inhaled hypnotic agents used. In particular, the CD56loCD16^+^ subset of NK cells, described as human adaptive-like NK cells (21), are known to express BNC2 (21–23) and to produce high amounts of IFN-γ (24), indicating they may be particularly susceptible. In line with these findings, an evaluation of the *in vitro* effects of sevoflurane on NK cells and their subsets revealed the greatest functional impact within the highly cytotoxic CD56loCD16^+^ NK cell population after 4 h of *in vitro* exposure to sevoflurane (20). Despite its expression in NK cells, the functional role of BNC2 in NK cell development, activation, cytotoxicity, or cytokine production remains undocumented and warrants further investigation.

We did observe significant alterations in cell number following gas induction, likely due to the redistribution of cells between peripheral blood and tissues, including secondary lymphoid organs (25). This effect was particularly evident in the absolute count of NK cells, which increased after induction and was mirrored by elevated expression of BNC2 in whole blood transcriptional data, a gene enriched in NK cells. Thus, caution is warranted when interpreting cell count data derived from whole blood. In contrast, the frequency of the vast majority of immune cell types remained unchanged following gas induction, including T helper cells, B-cell maturation subsets, regulatory T cells, and innate immune cell populations such as neutrophils, eosinophils, and dendritic cells. Although some small changes were observed after gas induction, these were minor compared to the variation observed between individuals of very similar ages.

Unbiased phenotyping of cellular responses at single-cell resolution is increasingly used to dissect complex responses to antigenic stimuli in both health and disease. Using single-cell transcriptional profiling, we demonstrate that similar phenotypes were observed before and after exposure to VAs. As these samples were derived from healthy individuals, we used the oligoclonal activator SEB to ensure a broad range of cellular phenotypes were represented, including naïve and memory T cells, without bias toward any particular helper cell subset. Although this does not represent a true antigen-specific response, the data suggest that the brief exposure to VAs is unlikely to have a major effect on the transcriptional profile of responding cells.

This study has several limitations that should be acknowledged. First, the sample size was relatively small (*n* = 10), and participants were limited to a narrow age range (3–6 years). While this allowed us to focus on an understudied cohort of young, healthy children, the findings may not be generalizable to infants, older children, or the broader pediatric populations. Second, the absence of a nonanesthetized control group means we cannot fully exclude the potential confounding effects of perioperative stressors, such as cannulation or surgery, although similar stressors are likely to be present in study populations undergoing sampling (e.g., individuals recently diagnosed with an immune-mediated disease). Third, although we observed transcriptional changes suggestive of innate immune activation, we were unable to perform functional validation (e.g., cytokine secretion or phagocytosis assays) due to limited blood volumes. Finally, we did not assess antigen-presenting cell (APC) function in detail; however, major shifts in APC activity would likely have been reflected in the bulk RNA sequencing data. These limitations underscore the need for future studies involving larger, more diverse cohorts and integrated functional analyses to further elucidate the impact of anesthetic agents on the immune system.

In summary, we demonstrate that blood samples taken under general anesthesia can be used to characterize the immune system composition and transcriptional profile of whole blood immune cells. However, a few caveats should be considered, particularly with respect to absolute cell numbers. In addition, these blood samples are also well suited for functional studies, including single-cell transcriptional profiling of activated CD4^+^ T cells (whether through polyclonal or antigen-specific stimulation), and offer a unique opportunity to recruit healthy children for research studies.

## Data Availability

The original contributions presented in the study are publicly available. This data can be found here: NCBI GEO, accession GSE303339.

## Members of the EXE-T1D consortium

Department of Clinical and Biomedical Sciences, University of Exeter Medical School, Exeter, United Kingdom: Amber M. Luckett, Andrew T. Hattersley, Kathryn Murrall, Matthew B. Johnson, Michelle Hudson, Noel G. Morgan, Rebecca A. Dobbs, Richard A. Oram, Sarah J. Richardson, Timothy J. McDonald. Department of Immunobiology, School of Immunology & Microbial Sciences (SIMS), King’s College London, United Kingdom: Clara Domingo-Vila, Timothy I. Tree. Center for Interventional Immunology, Benaroya Research Institute, Seattle, WA, United States: Cate Speake, Megan E. Smithmyer. Department of Internal Medicine, Leiden University Medical Center, Leiden, the Netherlands: Bart O. Roep. Department of Pediatrics, Indiana University School of Medicine, Indianapolis, IN, United States: William A Hagopian. Translational Health Sciences, Bristol Medical School, University of Bristol, Southmead Hospital, Bristol, United Kingdom: Kathleen M. Gillespie. Steering committee participant representative: Mendy Korer.
