# Supplementary material for: Blood samples collected under anesthesia can be used as a source of non-diseased controls for immune-based assays
Source: Front Immunol. 2025 Jul 24;16:1618080. doi: 10.3389/fimmu.2025.1618080 (PMC12330393; doi:10.3389/fimmu.2025.1618080)
Supplement: Supplementary file 1 [file DataSheet1.docx]

1. **Supplementary material**

Supplementary Table 1. Monoclonal antibodies used in the T cell flow cytometry panel

| **Marker (surface)** | **Fluorochrome** | **Clone** | **Company** |
| --- | --- | --- | --- |
| CD3 | APC-Cy7 | OKT3 | BioLegend |
| CD4 | BUV395 | SK3 | BD Biosciences |
| CD25 | BB515 | 2A3 | BD Biosciences |
| CD25 | BB515 | M-A251 | BD Biosciences |
| CD127 | APC | A019D5 | BioLegend |
| CD45RA | BV785 | HI100 | BioLegend |
| CD197/CCR7 | BV421 | G043H7 | BioLegend |
| CD183/CXCR3 | BV510 | G025H7 | BioLegend |
| CD194/CCR4 | BV605 | L291H4 | BioLegend |
| CD196/CCR6 | BUV737 | 11A9 | BD Biosciences |
| CD95 | PE | DX2 | BioLegend |
| CCR10 | PerCP-Cy5.5 | 1B5 | BD Biosciences |
| CD185/CXCR5 | PE-Cy7 | J252D4 | BioLegend |
| CD278/ICOS | BV711 | DX29 | BD Biosciences |
| CD279/PD-1 | PE-Dazzle 594 | EH12.2H7 | BioLegend |
| Brilliant stain buffer | N/A | N/A | BD Biosciences |

Supplementary Table 2. Monoclonal antibodies used in the B/DC/Monocyte flow cytometry panel

| **Marker (surface)** | **Fluorochrome** | **Clone** | **Company** |
| --- | --- | --- | --- |
| CD3 | APC-Cy7 | OKT3 | BioLegend |
| CD19 | PE | HIB19 | BioLegend |
| CD14 | PerCP-Cy5.5 | HCD14 | BioLegend |
| CD16 | PE-Cy7 | 3G8 | BioLegend |
| CD56 | APC | HCD56 | BioLegend |
| HLA-DR | FITC | LN3 | BioLegend |
| CD123 | BUV395 | 7G3 | BD Biosciences |
| CD11c | BV421 | Bu15 | BioLegend |
| IgD | BV605 | IA6-2 | BioLegend |
| CD27 | BUV737 | L128 | BD Biosciences |
| CD38 | BV785 | HIT2 | BioLegend |
| CD24 | BV510 | ML5 | BioLegend |
| Brilliant stain buffer | N/A | N/A | BD Biosciences |

Supplementary Table 3. Monoclonal antibodies used in the Granulocyte flow cytometry panel

| **Marker (surface)** | **Fluorochrome** | **Clone** | **Company** |
| --- | --- | --- | --- |
| CD45 | PerCP | HI30 | BioLegend |
| CD3 | APC-Cy7 | OKT3 | BioLegend |
| CD19 | APC-Cy7 | HIB19 | BioLegend |
| CD56 | APC-Cy7 | HCD56 | BioLegend |
| CD14 | AF488 | HCD14 | BioLegend |
| CD15 | BV605 | W6D3 | BioLegend |
| CD64 | BV421 | 10.1 | BioLegend |
| CD63 | APC | HC56 | BioLegend |
| CD123 | BUV395 | 7G3 | BD Biosciences |
| CD294 | PE | BM16 | Biolegend |
| CD203c | BV510 | NP4D6 | BioLegend |
| CD69 | PE-Cy7 | FN50 | BioLegend |
| Brilliant stain buffer | N/A | N/A | BD Biosciences |

Supplementary Table 4. Monoclonal antibodies used in the Lineage flow cytometry panel

| **Marker (surface)** | **Fluorochrome** | **Clone** | **Company** |
| --- | --- | --- | --- |
| CD45 | PerCP | HI30 | BioLegend |
| CD3 | APC-Cy7 | OKT3 | BioLegend |
| CD4 | BUV395 | SK3 | BD Biosciences |
| CD8 | BUV737 | SK1 | BD Biosciences |
| CD19 | PE | HIB19 | BioLegend |
| CD14 | AF488 | HCD14 | BioLegend |
| CD16 | PE-Cy7 | 3G8 | BioLegend |
| CD15 | BV605 | W6D3 | BioLegend |
| CD56 | APC | HCD56 | BioLegend |
| Brilliant stain buffer | N/A | N/A | BD Biosciences |

Supplementary Table 5. Monoclonal antibodies used in the Treg flow cytometry panel

| **Marker (surface)** | **Fluorochrome** | **Clone** | **Company** |
| --- | --- | --- | --- |
| CD45RA | BV785 | HI100 | BioLegend |
| **Marker (intracellular)** | **Fluorochrome** | **Clone** | **Company** |
| CD15s | BV510 | CSLEX1 | BD Biosciences |
| CD3 | BV605 | OKT3 | BioLegend |
| CD4 | BUV395 | SK3 | BD Biosciences |
| CD8 | BUV737 | SK1 | BD Biosciences |
| CD25 | PE | M-A251 | BD Biosciences |
| FOXP3 | AF647 | 259D | Beckman Coulter |
| Helios | Pacific Blue | 22F6 | BioLegend |
| Ki67 | FITC | B56 | BD Biosciences |
| CD69 | PE-Cy7 | FN50 | BioLegend |

**Supplementary Table 6. Monoclonal antibodies used for single-cell RNA-sequencing analysis**

| **Marker (surface)** | **Fluorochrome/**  **Barcode Sequence** | **Clone** | **Company** |
| --- | --- | --- | --- |
| CD4 | Alexa Fluor 700 | OKT4 | BioLegend |
| CD137 (4-1BB) | BV421 | 4B4-1 | BioLegend |
| CD8 | PE-Cy7 | RPA-T8 | BioLegend |
| GARP | eFluor 660 | G14D9 | BioLegend |
| CD19 | APC-Cy7 | HIB19 | BioLegend |
| CD14 | APC-Cy7 | HCD14 | BioLegend |
| CD69 | FITC | FN50 | BioLegend |
| CD154 | BV711 | 24-31 | BioLegend |
| CD45RA | BV785 | HI100 | BioLegend |
| TotalSeq-CD137 | CAGTAAGTTCGGGAC | 4B4-1 | BioLegend |
| TotalSeq-CD154 | GCTAGATAGATGCAA | 24-31 | BioLegend |
| TotalSeq-CD69 | GTCTCTTGGCTTAAA | FN50 | BioLegend |
| TotalSeq-GARP | AGGTATGGTAGAGTA | 7B11 | BioLegend |
| TotalSeq-CD26 | GGTGGCTAGATAATG | BA5b | BioLegend |
| TotalSeq-CD319 | AGTATGCCATGTCTT | 162.1 | BioLegend |
| TotalSeq-CD57 | AACTCCCTATGGAGG | QA17A04 | BioLegend |
| TotalSeq-CD314 | CGTGTTTGTTCCTCA | 1D11 | BioLegend |
| TotalSeq-CD4 | GAGGTTAGTGATGGA | SK3 | BioLegend |
| TotalSeq-CD94 | CTTTCCGGTCCTACA | DX22 | BioLegend |
| Brilliant stain buffer Plus | N/A | N/A | BD Biosciences |

**Supplementary Table 7. Summary table of multiple paired t tests performed on flow cytometry data.**

| **Cell population** | **Mean of**  **Pre-SEVO** | **Mean of**  **Post-SEVO** | **P value** | **Adjusted**  **P Value** |
| --- | --- | --- | --- | --- |
| % True naïve (Teff) | 73.32 | 72.4 | 0.080296 | >0.999999 |
| % Teff Tscm (Teff) | 2.429 | 2.356 | 0.292224 | >0.999999 |
| % Teff Tcm (Teff) | 16.77 | 16.52 | 0.269625 | >0.999999 |
| % Teff Tem (Teff) | 6.148 | 6.969 | 0.001022 | 0.102185 |
| % Teff Temra (Teff) | 1.324 | 1.744 | 0.169464 | >0.999999 |
| % Teff Th1 (mTeff) | 12.71 | 13.66 | 0.283981 | >0.999999 |
| % Teff Th1Th17 (mTeff) | 5.356 | 5.666 | 0.108419 | >0.999999 |
| % Teff Th2 (mTeff) | 7.579 | 7.228 | 0.345899 | >0.999999 |
| % Teff Th17 (mTeff) | 7.594 | 7.578 | 0.966686 | >0.999999 |
| % Teff Th22 (mTeff) | 1.815 | 1.862 | 0.520286 | >0.999999 |
| % Th1Th2 (mTeff) | 21.54 | 21.1 | 0.676938 | >0.999999 |
| % True naïve (Treg) | 53.66 | 51.94 | 0.177445 | >0.999999 |
| % Treg Tscm (Treg) | 7.429 | 7.494 | 0.688494 | >0.999999 |
| % Treg Tcm (Treg) | 21.9 | 21.74 | 0.644542 | >0.999999 |
| % Treg Tem (Treg) | 15.62 | 16.55 | 0.01519 | >0.999999 |
| % Treg Temra (Treg) | 1.357 | 1.332 | 0.839107 | >0.999999 |
| % Treg Th1 (mTreg) | 5.791 | 5.966 | 0.677414 | >0.999999 |
| % Treg Th1Th17 (mTreg) | 0.651 | 0.713 | 0.481504 | >0.999999 |
| % Treg Th2 (mTreg) | 13.32 | 12.76 | 0.476267 | >0.999999 |
| % Treg Th17 (mTreg) | 11.2 | 10.39 | 0.139215 | >0.999999 |
| % Treg Th22 (mTreg) | 8.263 | 9.032 | 0.197727 | >0.999999 |
| % Th1Th2 (mTreg) | 27.9 | 26.77 | 0.086312 | >0.999999 |
| % True naïve (CD8) | 43.34 | 37.84 | 0.000591 | 0.05908 |
| % CD8 Tscm (CD8) | 4.012 | 3.452 | 0.000843 | 0.084349 |
| % CD8 Tcm (CD8) | 2.616 | 2.593 | 0.855898 | >0.999999 |
| % CD8 Tem (CD8) | 12.07 | 13.08 | 0.14318 | >0.999999 |
| % CD8 Temra (CD8) | 27.84 | 33.79 | 0.001934 | 0.193444 |
| % CD25hiCD127loTregs (CD4) | 8.718 | 8.755 | 0.704074 | >0.999999 |
| % Teff Tfh (mTeff) | 37.24 | 36.23 | 0.658561 | >0.999999 |
| % ICOS- PD-1+ (Teff Tfh) | 15.28 | 16.5 | 0.006788 | 0.678779 |
| % ICOS+ PD-1+ (Teff Tfh) | 4.906 | 5.193 | 0.204594 | >0.999999 |
| % ICOS+ PD-1- (Teff Tfh) | 6.66 | 5.856 | 0.269444 | >0.999999 |
| % CCR6- CXCR3+ (Teff Tfh) | 37.28 | 37.25 | 0.952749 | >0.999999 |
| % CCR6+ CXCR3+ (Teff Tfh) | 11.26 | 11.23 | 0.964967 | >0.999999 |
| % CCR6+ CXCR3- (Teff Tfh) | 19.65 | 20.15 | 0.161699 | >0.999999 |
| % Treg Tfh (mTreg) | 21.91 | 22.71 | 0.783192 | >0.999999 |
| % ICOS- PD-1+ (Treg Tfh) | 10.77 | 10.7 | 0.981488 | >0.999999 |
| % ICOS+ PD-1+ (Treg Tfh) | 10.36 | 12.76 | 0.089711 | >0.999999 |
| % ICOS+ PD-1- (Treg Tfh) | 17.02 | 17 | 0.992505 | >0.999999 |
| % CCR6- CXCR3+ (Treg Tfh) | 26.56 | 25.96 | 0.782677 | >0.999999 |
| % CCR6+ CXCR3+ (Treg Tfh) | 9.159 | 8.294 | 0.473423 | >0.999999 |
| % CCR6+ CXCR3- (Treg Tfh) | 21.88 | 24.63 | 0.474878 | >0.999999 |
| % ICOS- PD-1+ (CXCR5- Teff) | 12.04 | 12.38 | 0.377981 | >0.999999 |
| % ICOS+ PD-1+ (CXCR5- Teff) | 3.11 | 3.101 | 0.935068 | >0.999999 |
| % ICOS+ PD-1- (CXCR5- Teff) | 7.028 | 6.801 | 0.309274 | >0.999999 |
| % ICOS- PD-1+ (CXCR5- Treg) | 6.16 | 6.604 | 0.100339 | >0.999999 |
| % ICOS+ PD-1+ (CXCR5- Treg) | 6.483 | 6.426 | 0.866315 | >0.999999 |
| % ICOS+ PD-1- (CXCR5- Treg) | 19.15 | 17.51 | 0.137022 | >0.999999 |
| % DC (MNC) | 0.726 | 0.826 | 0.077979 | >0.999999 |
| % Myeloid DC (DC) | 45.05 | 42.75 | 0.61127 | >0.999999 |
| % pDC (DC) | 52.54 | 54.49 | 0.66583 | >0.999999 |
| % Plasmablast (B) | 4.186 | 4.174 | 0.958391 | >0.999999 |
| % Circulating B (B) | 8.608 | 8.453 | 0.345189 | >0.999999 |
| % Classical memory B (B) | 11.39 | 11.23 | 0.546509 | >0.999999 |
| % Double negative B (B) | 6.323 | 7.309 | 0.020036 | >0.999999 |
| % Naive B (B) | 69.48 | 68.86 | 0.225124 | >0.999999 |
| % Transitional B (B) | 10.19 | 9.328 | 0.048163 | >0.999999 |
| % CD56hi NK (NK) | 6.903 | 3.534 | 0.018469 | >0.999999 |
| % CD56loCD16+ NK (NK) | 87.42 | 93.48 | 0.009708 | 0.970801 |
| % CD56loCD16- NK (NK) | 2.997 | 1.758 | 0.002294 | 0.229448 |
| % CD56hiCD16+ NK (NK) | 2.68 | 1.228 | 0.031902 | >0.999999 |
| % CD14+ Monocyte (monocyte) | 79.43 | 72.49 | 0.004653 | 0.465338 |
| % CD14+CD16+ Monocyte (monocyte) | 4.174 | 5.347 | 0.018598 | >0.999999 |
| % CD16+ Monocyte (monocyte) | 16.38 | 22.11 | 0.015266 | >0.999999 |
| % CD4+ Teff CD15s+ (Teff) | 0.888 | 0.874 | 0.717412 | >0.999999 |
| % CD4+ Teff CD69+ (Teff) | 1.788 | 1.791 | 0.980033 | >0.999999 |
| % CD4+ Teff Ki67+ (Teff) | 2.241 | 2.204 | 0.626143 | >0.999999 |
| % CD25+FOXP3+Treg(CD4) | 7.603 | 7.667 | 0.672794 | >0.999999 |
| % aTreg Foxp3hi (Treg) | 7.912 | 8.523 | 0.237509 | >0.999999 |
| % mTreg Foxp3lo (Treg) | 31.61 | 31.47 | 0.831269 | >0.999999 |
| % rTreg Foxp3lo naive (Treg) | 59.6 | 59.25 | 0.597961 | >0.999999 |
| % CD15s+ Treg (Treg) | 2.903 | 2.779 | 0.466515 | >0.999999 |
| % CD69+ Treg (Treg) | 3.226 | 3.267 | 0.785956 | >0.999999 |
| % Helios+ Treg (Treg) | 84.96 | 83.96 | 0.233252 | >0.999999 |
| % Ki67+ Treg (Treg) | 7.059 | 7.501 | 0.148004 | >0.999999 |
| % CD15s+ CD8 (CD8) | 0.58 | 0.569 | 0.787718 | >0.999999 |
| % CD69+ CD8 (CD8) | 3.224 | 3.846 | 0.01074 | >0.999999 |
| % Helios+ CD8 (CD8) | 21.73 | 22.84 | 0.570641 | >0.999999 |
| % Ki67+ CD8 (CD8) | 1.852 | 1.866 | 0.858228 | >0.999999 |
| % FOXP3+ CD8 (CD8) | 0.242 | 0.2486 | 0.832129 | >0.999999 |
| % CD4+ CD25-FOXP3+ (CD4) | 1.114 | 1.208 | 0.03042 | >0.999999 |
| % CD4+ CD25-FOXP3+ Helios+ (CD25-FOXP3+) | 64.06 | 65 | 0.482006 | >0.999999 |
| % CD4+ CD25-FOXP3+ Ki67+ (CD25-FOXP3+) | 11.71 | 11.69 | 0.98052 | >0.999999 |
| % Eosinophil (WBC, CD45+) | 2.132 | 2.139 | 0.967365 | >0.999999 |
| % Neutrophil (WBC, CD45+) | 45.23 | 43.14 | 0.369039 | >0.999999 |
| % Basophil (WBC, CD45+) | 0.424 | 0.418 | 0.887228 | >0.999999 |
| % Eosinophil CD69+ (eosinophil) | 14.18 | 14.7 | 0.430598 | >0.999999 |
| % Neutrophil CD69+ (neutrophil) | 11.22 | 12.24 | 0.518325 | >0.999999 |
| % Basophil CD69+ (basophil) | 14.63 | 14.38 | 0.884985 | >0.999999 |
| % CD3+ (WBC, CD45+) | 35.04 | 32.21 | 0.269097 | >0.999999 |
| % CD3+CD56- T (WBC, CD45+) | 34.63 | 31.75 | 0.262519 | >0.999999 |
| % NK (WBC, CD45+) | 3.189 | 6.626 | 0.000717 | 0.071662 |
| % Monocyte (WBC, CD45+) | 4.653 | 5.432 | 0.097059 | >0.999999 |
| % CD19+ B (WBC, CD45+) | 11.36 | 11.21 | 0.719311 | >0.999999 |
| % CD4+ T (CD3+T) | 55.74 | 52.23 | 0.000034 | 0.003362 |
| % CD4+CD8+ T (CD3+T) | 0.3122 | 0.3347 | 0.341923 | >0.999999 |
| % CD8+ T (CD3+T) | 32.84 | 35.08 | 0.001915 | 0.191466 |
| % CD56+ NKT (WBC, CD45+) | 0.412 | 0.469 | 0.009742 | 0.974184 |
| % CD4+CD56+ NKT(NKT) | 7.683 | 6.487 | 0.13311 | >0.999999 |
| % CD8+CD56+ NKT (NKT) | 66.09 | 68.32 | 0.022408 | >0.999999 |

*Note*: Threshold for adjusted p value is alpha = 0.05.

**Supplementary Table 8. Ratios between variance explained by SEVO effect and variance explained by individuals.**

| **Cell population** | **Ratio** | **Low CI** | **Up CI** |
| --- | --- | --- | --- |
| % True naïve (Teff) | 0.0068 | 0.0001 | 0.0255 |
| % Teff Tscm (Teff) | 0.0004 | 0.0000 | 0.0021 |
| % Teff Tcm (Teff) | 0.0004 | 0.0000 | 0.0026 |
| % Teff Tem (Teff) | 0.0206 | 0.0073 | 0.0366 |
| % Teff Temra (Teff) | 0.0082 | 0.0001 | 0.0384 |
| % Teff Th1 (mTeff) | 0.0027 | 0.0000 | 0.0159 |
| % Teff Th1Th17 (mTeff) | 0.0023 | 0.0001 | 0.0085 |
| % Teff Th2 (mTeff) | 0.0035 | 0.0000 | 0.0312 |
| % Teff Th17 (mTeff) | 0.0000 | 0.0000 | 0.0468 |
| % Teff Th22 (mTeff) | 0.0015 | 0.0000 | 0.0191 |
| % Th1Th2 (mTeff) | 0.0014 | 0.0000 | 0.0327 |
| % True naïve (Treg) | 0.0526 | 0.0005 | 0.2166 |
| % Treg Tscm (Treg) | 0.0000 | 0.0000 | 0.0012 |
| % Treg Tcm (Treg) | 0.0001 | 0.0000 | 0.0020 |
| % Treg Tem (Treg) | 0.0065 | 0.0008 | 0.0161 |
| % Treg Temra (Treg) | 0.0000 | 0.0000 | 0.0039 |
| % Treg Th1 (mTreg) | 0.0017 | 0.0000 | 0.0430 |
| % Treg Th1Th17 (mTreg) | 0.0070 | 0.0001 | 0.0674 |
| % Treg Th2 (mTreg) | 0.0019 | 0.0000 | 0.0184 |
| % Treg Th17 (mTreg) | 0.0152 | 0.0002 | 0.0631 |
| % Treg Th22 (mTreg) | 0.0049 | 0.0001 | 0.0215 |
| % Th1Th2 (mTreg) | 0.0039 | 0.0001 | 0.0142 |
| % True naïve (CD8) | 0.0675 | 0.0264 | 0.1225 |
| % CD8 Tscm (CD8) | 0.0205 | 0.0079 | 0.0389 |
| % CD8 Tcm (CD8) | 0.0000 | 0.0000 | 0.0014 |
| % CD8 Tem (CD8) | 0.0050 | 0.0001 | 0.0203 |
| % CD8 Temra (CD8) | 0.0775 | 0.0263 | 0.1438 |
| % CD25hiCD127loTregs (CD4) | 0.0002 | 0.0000 | 0.0071 |
| % Teff Tfh (mTeff) | 0.0009 | 0.0000 | 0.0241 |
| % ICOS- PD-1+ (Teff Tfh) | 0.0052 | 0.0013 | 0.0119 |
| % ICOS+ PD-1+ (Teff Tfh) | 0.0105 | 0.0001 | 0.0498 |
| % ICOS+ PD-1- (Teff Tfh) | 0.0348 | 0.0000 | 0.1631 |
| % CCR6- CXCR3+ (Teff Tfh) | 0.0000 | 0.0000 | 0.0092 |
| % CCR6+ CXCR3+ (Teff Tfh) | 0.0000 | 0.0000 | 0.0162 |
| % CCR6+ CXCR3- (Teff Tfh) | 0.0036 | 0.0001 | 0.0157 |
| % Treg Tfh (mTreg) | 0.0004 | 0.0000 | 0.0227 |
| % ICOS- PD-1+ (Treg Tfh) | 0.0001 | 0.0001 | 0.1870 |
| % ICOS+ PD-1+ (Treg Tfh) | 0.0189 | 0.0003 | 0.0682 |
| % ICOS+ PD-1- (Treg Tfh) | 0.0000 | 0.0001 | 0.0828 |
| % CCR6- CXCR3+ (Treg Tfh) | 0.0024 | 0.0002 | 0.0870 |
| % CCR6+ CXCR3+ (Treg Tfh) | 0.0107 | 0.0002 | 0.0901 |
| % CCR6+ CXCR3- (Treg Tfh) | Inf | 0.0002 | Inf |
| % ICOS- PD-1+ (CXCR5- Teff) | 0.0003 | 0.0000 | 0.0024 |
| % ICOS+ PD-1+ (CXCR5- Teff) | 0.0000 | 0.0000 | 0.0052 |
| % ICOS+ PD-1- (CXCR5- Teff) | 0.0010 | 0.0000 | 0.0072 |
| % ICOS- PD-1+ (CXCR5- Treg) | 0.0015 | 0.0001 | 0.0061 |
| % ICOS+ PD-1+ (CXCR5- Treg) | 0.0000 | 0.0000 | 0.0060 |
| % ICOS+ PD-1- (CXCR5- Treg) | 0.0150 | 0.0002 | 0.0603 |
| % DC (MNC) | 0.2079 | 0.0032 | 0.5330 |
| % Myeloid DC (DC) | 0.0636 | 0.0007 | 1.3127 |
| % pDC (DC) | 0.0445 | 0.0003 | 1.0446 |
| % Plasmablast (B) | 0.0000 | 0.0000 | 0.0015 |
| % Circulating B (B) | 0.0008 | 0.0000 | 0.0066 |
| % Classical memory B (B) | 0.0006 | 0.0000 | 0.0080 |
| % Double negative B (B) | 0.0605 | 0.0100 | 0.1498 |
| % Naive B (B) | 0.0018 | 0.0000 | 0.0097 |
| % Transitional B (B) | 0.0119 | 0.0006 | 0.0343 |
| % CD56hi NK (NK) | 0.4471 | 0.0362 | 0.8598 |
| % CD56loCD16+ NK (NK) | 0.5562 | 0.0704 | 1.0088 |
| % CD56loCD16- NK (NK) | 0.2940 | 0.0680 | 0.4617 |
| % CD56hiCD16+ NK (NK) | 0.2329 | 0.0215 | 0.4664 |
| % CD14+ Monocyte (monocyte) | 0.0383 | 0.0096 | 0.0777 |
| % CD14+CD16+ Monocyte (monocyte) | 0.0738 | 0.0107 | 0.1818 |
| % CD16+ Monocyte (monocyte) | 0.0228 | 0.0041 | 0.0558 |
| % CD4+ Teff CD15s+ (Teff) | 0.0003 | 0.0000 | 0.0088 |
| % CD4+ Teff CD69+ (Teff) | 0.0000 | 0.0000 | 0.0243 |
| % CD4+ Teff Ki67+ (Teff) | 0.0002 | 0.0000 | 0.0036 |
| % CD25+FOXP3+Treg (CD4) | 0.0004 | 0.0000 | 0.0119 |
| % aTreg Foxp3hi (Treg) | 0.0414 | 0.0005 | 0.1887 |
| % mTreg Foxp3lo (Treg) | 0.0001 | 0.0000 | 0.0100 |
| % rTreg Foxp3lo naive (Treg) | 0.0005 | 0.0000 | 0.0089 |
| % CD15s+ Treg (Treg) | 0.0058 | 0.0001 | 0.0587 |
| % CD69+ Treg (Treg) | 0.0002 | 0.0000 | 0.0149 |
| % Helios+ Treg (Treg) | 0.0187 | 0.0002 | 0.0859 |
| % Ki67+ Treg (Treg) | 0.0245 | 0.0003 | 0.1039 |
| % CD15s+ CD8 (CD8) | 0.0003 | 0.0000 | 0.0160 |
| % CD69+ CD8 (CD8) | 0.4235 | 0.0771 | 0.6904 |
| % Helios+ CD8 (CD8) | 0.0065 | 0.0001 | 0.0654 |
| % Ki67+ CD8 (CD8) | 0.0000 | 0.0000 | 0.0064 |
| % FOXP3+ CD8 (CD8) | 0.0013 | 0.0001 | 0.1290 |
| % CD4+ CD25-FOXP3+ (CD4) | 0.0350 | 0.0036 | 0.0804 |
| % CD4+ CD25-FOXP3+ Helios+ (CD25-FOXP3+) | 0.0017 | 0.0000 | 0.0172 |
| % CD4+ CD25-FOXP3+ Ki67+ (CD25-FOXP3+) | 0.0000 | 0.0001 | 0.0600 |
| % Eosinophil (WBC, CD45+) | 0.0000 | 0.0001 | 0.0132 |
| % Neutrophil (WBC, CD45+) | 0.0041 | 0.0000 | 0.0261 |
| % Basophil (WBC, CD45+) | 0.0006 | 0.0002 | 0.0670 |
| % Eosinophil CD69+ (eosinophil) | 0.0055 | 0.0000 | 0.0515 |
| % Neutrophil CD69+ (neutrophil) | 0.0124 | 0.0001 | 0.1592 |
| % Basophil CD69+ (basophil) | 0.0023 | 0.0002 | 0.1900 |
| % CD3+ (WBC, CD45+) | 0.0128 | 0.0001 | 0.0714 |
| % CD3+CD56- T (WBC, CD45+) | 0.0134 | 0.0001 | 0.0746 |
| % NK (WBC, CD45+) | 1.6029 | 0.2420 | 1.9354 |
| % Monocyte (WBC, CD45+) | 0.0384 | 0.0006 | 0.1216 |
| % CD19+ B (WBC, CD45+) | 0.0002 | 0.0000 | 0.0052 |
| % CD4+ T (CD3+T) | 0.1063 | 0.0550 | 0.1582 |
| % CD4+CD8+ T (CD3+T) | 0.0013 | 0.0000 | 0.0097 |
| % CD8+ T (CD3+T) | 0.0793 | 0.0224 | 0.1478 |
| % CD56+ NKT (WBC, CD45+) | 0.0113 | 0.0025 | 0.0256 |
| % CD4+CD56+ NKT (NKT) | 0.0381 | 0.0009 | 0.1440 |
| % CD8+CD56+ NKT (NKT) | 0.0142 | 0.0020 | 0.0382 |

| 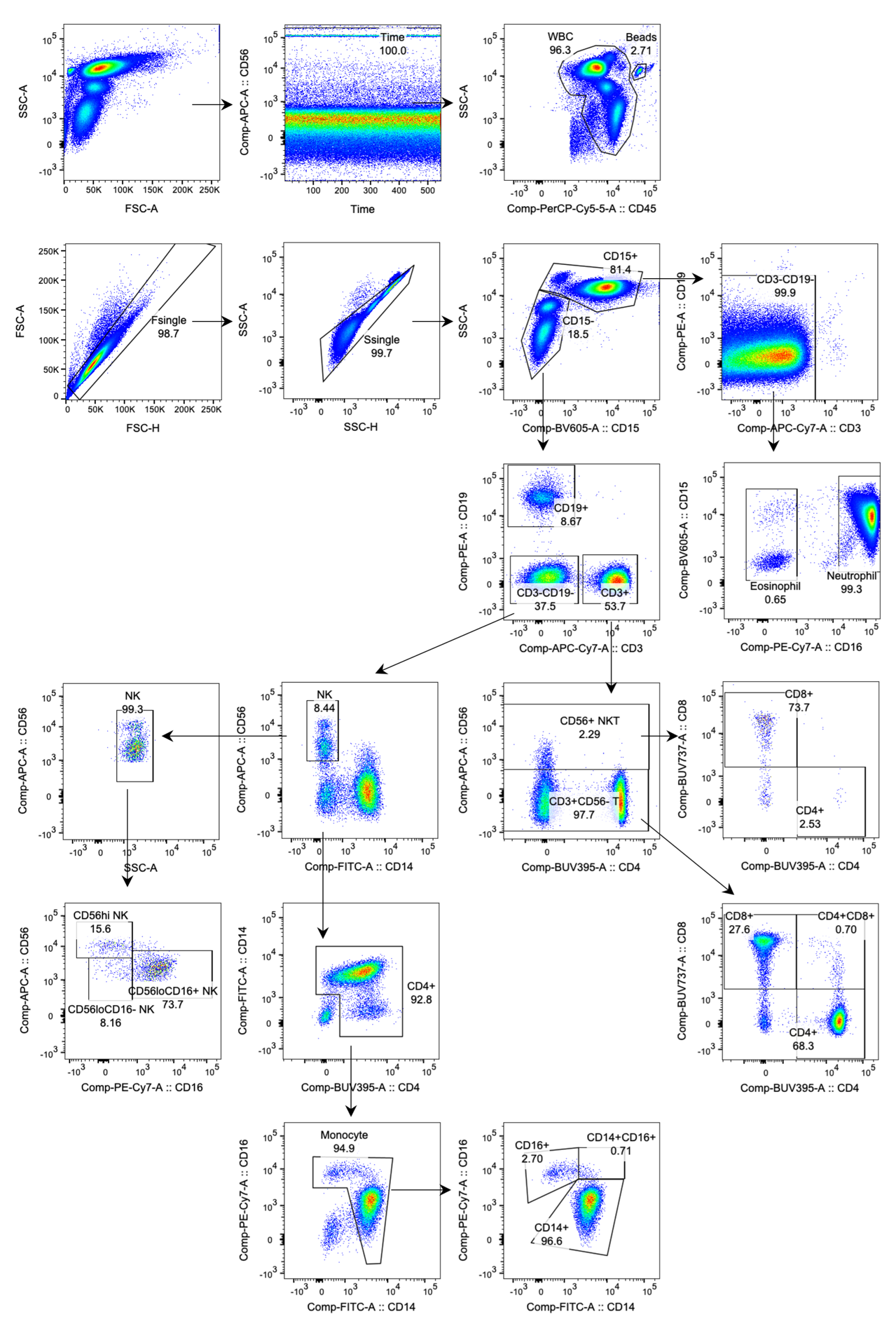 |
| --- |

**Supplementary Figure 1. Lineage panel gating strategy.** Events were first plotted versus time to exclude events acquired during periods of unstable flow. White blood cells (WBC) and beads were gated independently, and doublets and debris were excluded by gating on forward-scatter (FSC)-height versus FSC-area followed by side-scatter (SSC)-height and SSC-area. Expression of CD3, CD19, CD15 and CD16 were used to identify Eosinophils and Neutrophils, CD3+T and CD19+ B lymphocytes. CD4 and CD56 expression on CD3+T cells was used to distinguish CD56+NKT cells from CD3+CD56-T cells. Expression of CD4 and CD8 allowed identification of CD3+CD56-CD4+T, CD3+CD56-CD8+T, CD3+CD56-CD4+CD8+T, CD56+CD4+NKT, CD56+CD8+NKT cells. NK cells were identified as CD15-, CD3- and CD19-. Expression of CD56 and CD16 allowed identification of NK cell subsets; CD56loCD16+NK, CD56loCD16-NK, CD56hiNK and CD56hiCD16+NK. The ‘not gate’ function was used to determine monocytes and their classical, intermediate, and non-classical subsets by CD4, CD14 and CD16 expression. Gating from parental populations is assigned with arrows.

| 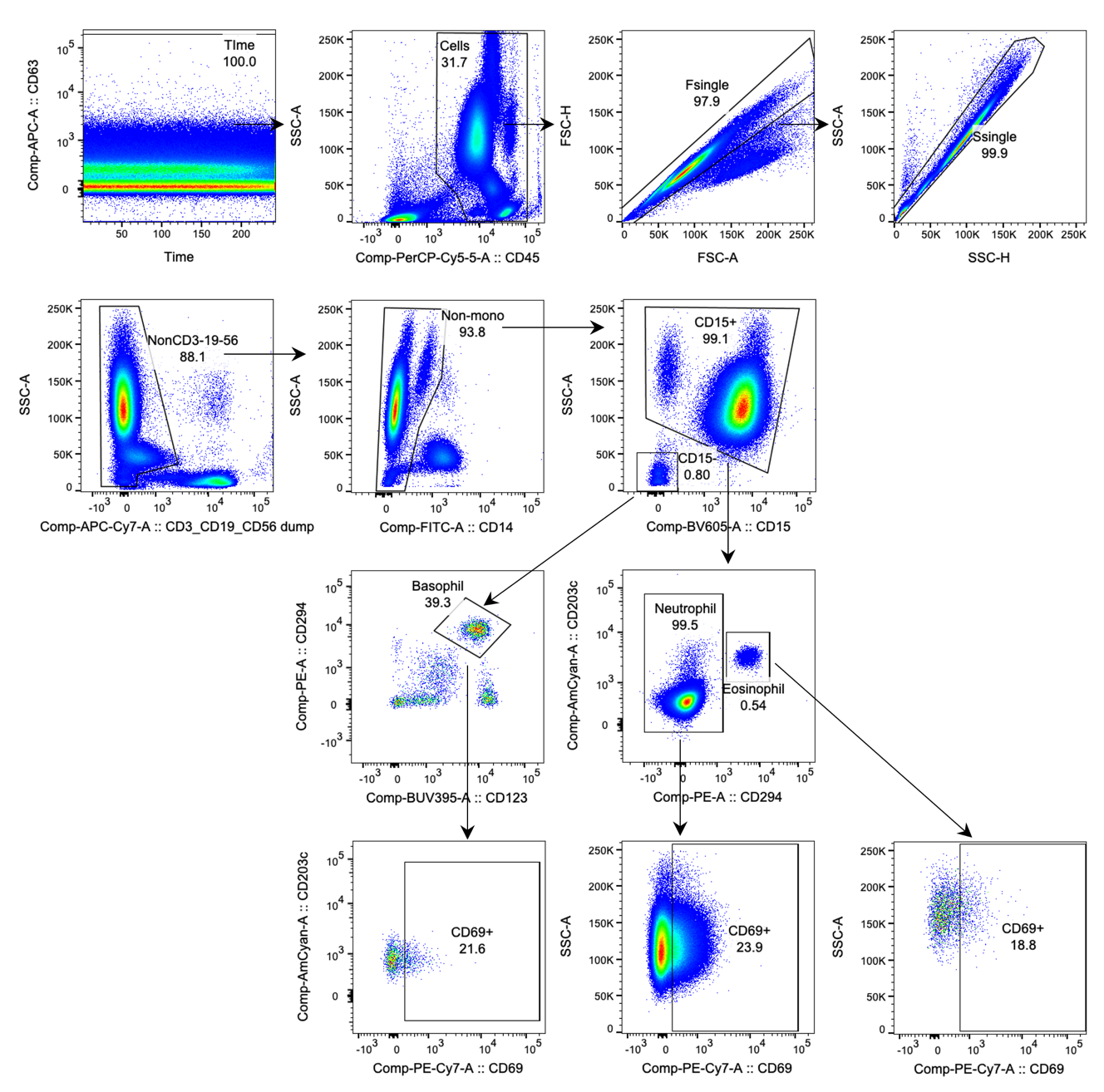 |
| --- |

**Supplementary Figure 2. Granulocyte panel gating strategy.** All cells were first gated across time. Leukocytes were gated based on SSC-area and CD45. Doublets and debris were excluded by gating on FSC-height and FSC-area followed by SSC-height and SSC-area. CD3, CD19 and CD56 were used as DUMP channel to exclude CD3+ and CD19+ lymphocytes and CD56 NK cells, and CD14 was used to exclude monocytes. CD15, CD294, CD203c and CD123 expression allowed identification of Neutrophils, Eosinophils and Basophils, respectively, and CD69 expression was used to determine an activated subset within these immune cell populations. Gating from parental populations is assigned with arrows.

| 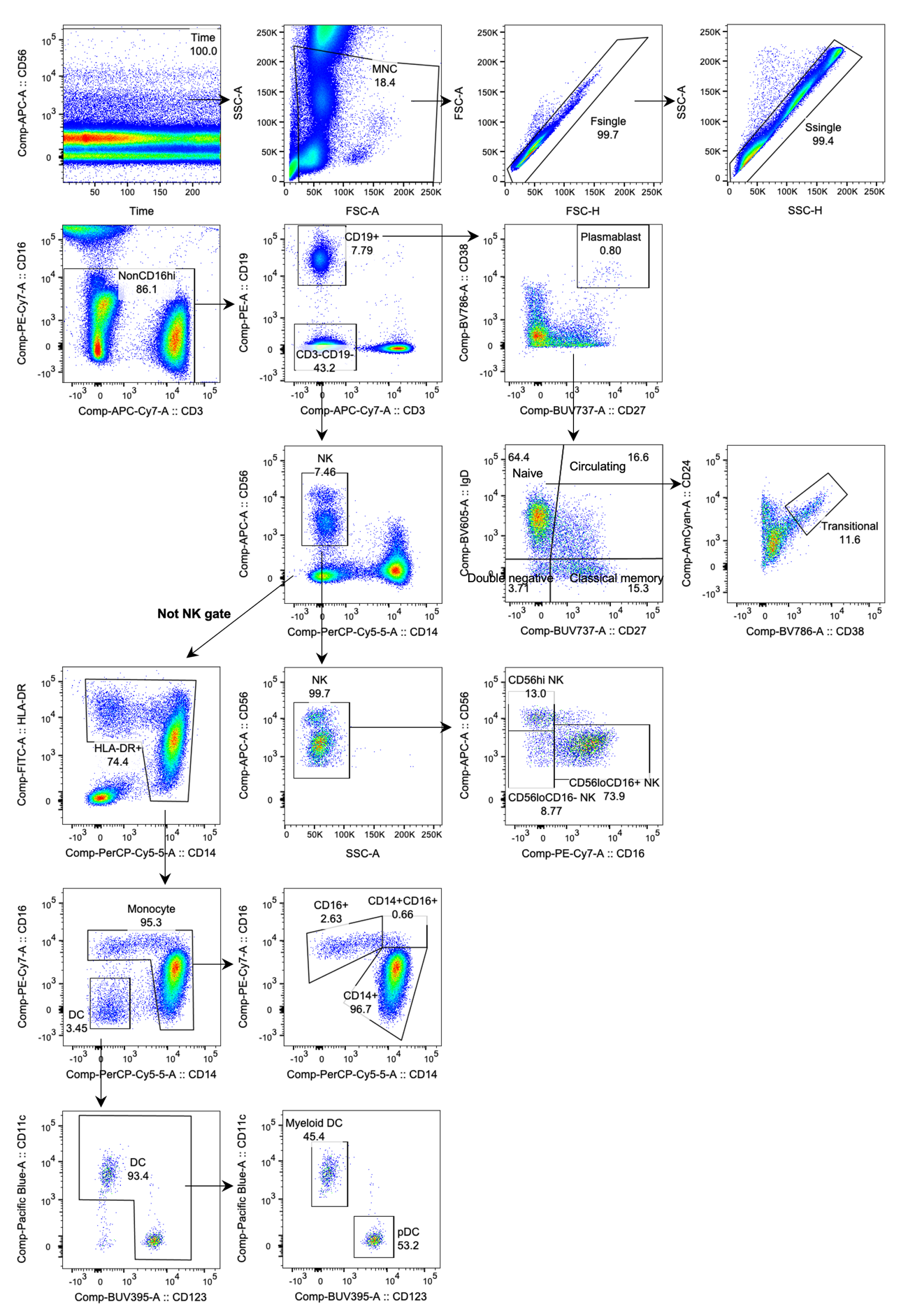 |
| --- |

**Supplementary Figure 3. B, DC, Monocyte and NK panel gating strategy.** All cells were first gated across time. Mononuclear cells (MNC) were gated based on FSC-area and SSC-area properties, followed by exclusion of doublets and debris by gating on FSC-height and FSC-area and by SSC-height and SSC-area. CD3, CD16 and CD19 expression was used to identify CD19+ B lymphocytes. Plasmablasts were defined as CD27 and CD38 positive whilst the naïve, circulating, classical memory and double negative subsets of CD19+ B cells were determined by CD27 and IgD expression, and for being negative for CD27 and CD38. Expression of CD38 and CD24 in naïve CD19+ B lymphocytes were used to define the transitional CD19+ B cell population. NK cells were identified as neither CD3 nor CD19. Expression of CD56 and CD16 allowed identification of NK cell subsets; CD56loCD16+NK, CD56loCD16-NK, CD56hiNK and CD56hiCD16+NK. The ‘not gate’ function was used to determine monocytes and DCs based on expression of HLADR and CD14. The classical, intermediate, and non-classical monocyte subsets were determined by CD4, CD14 and CD16 expression. The DC cell population was identified as neither CD14 nor CD16 and its subsets myeloid and plasmacytoid DCs were determined by CD123 and CD11c expression. Gating from parental populations is assigned with arrows.

| 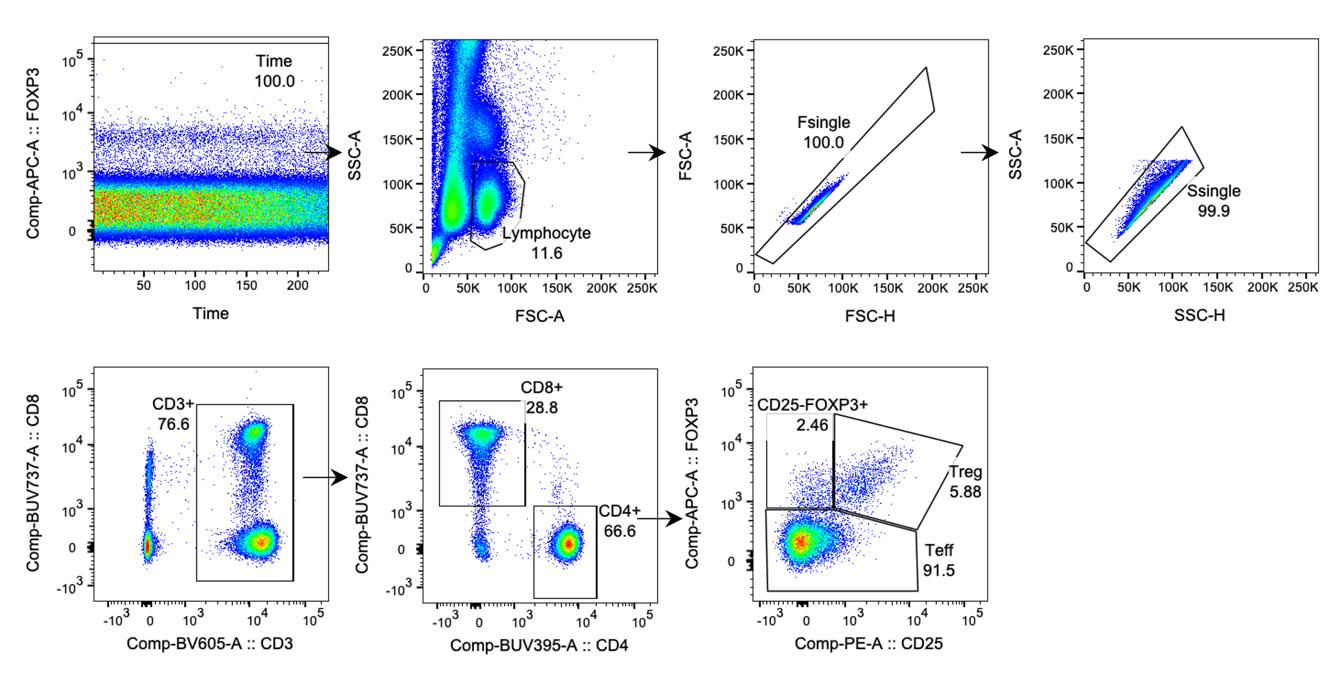 |
| --- |

**Supplementary Figure 4. Treg panel gating strategy.** All cells were first gated across time. Lymphocytes were gated based on FSC-area and SSC-area properties, followed by exclusion of doublets and debris by gating on FSC-height and FSC-area and by SSC-height and SSC-area. CD4+T and CD8+T lymphocytes were defined based on expression of CD3, CD4 and CD8. CD4+T cell subsets were determined based on expression of CD25 and FOXP3; CD4+Teff as CD4+CD25+/-FOXP3-, CD4+Treg as CD4+CD25+FOXP3+ and CD4+CD25-FOXP3+. Gating from parental populations is assigned with arrows.

| 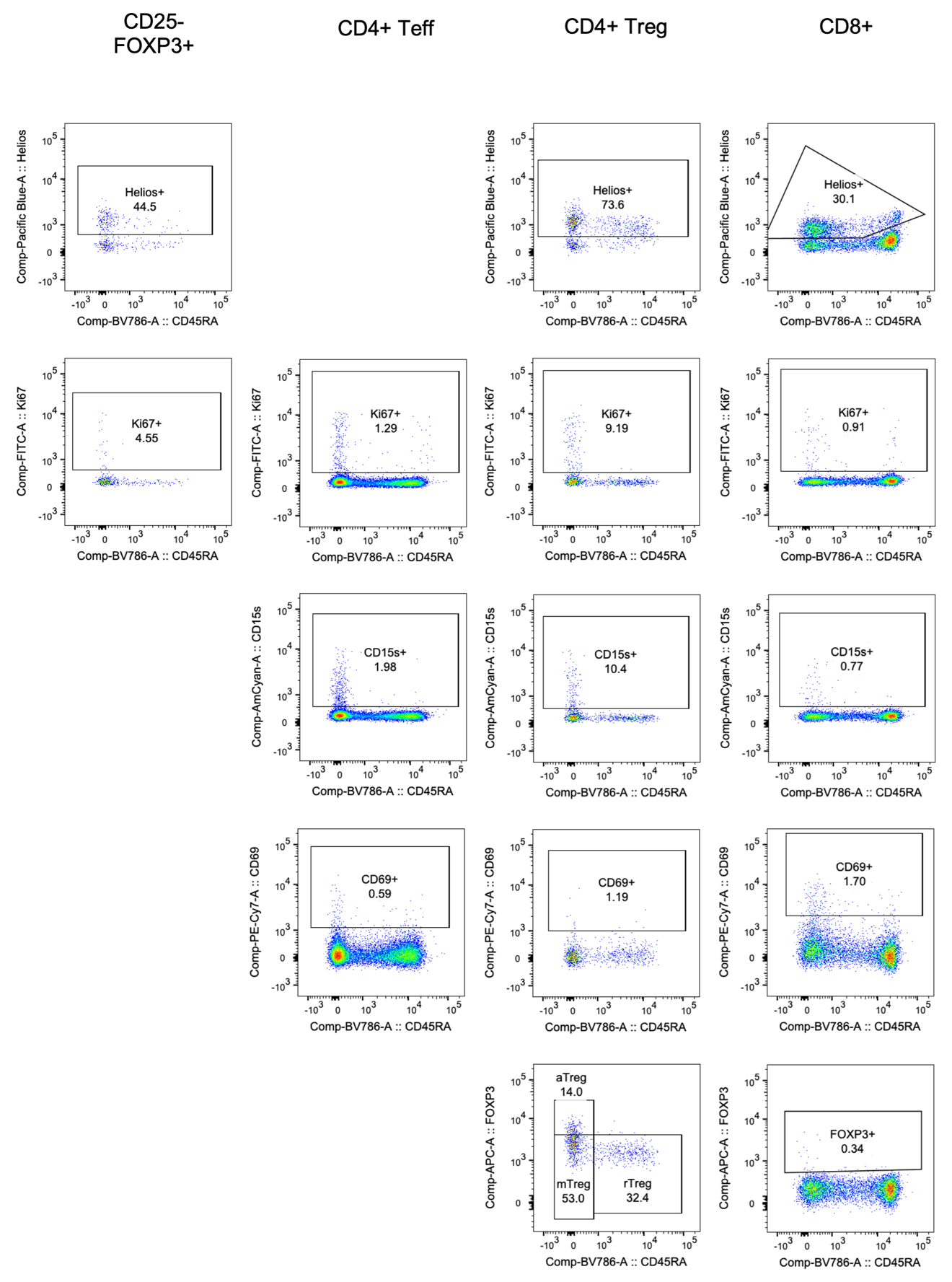 |
| --- |

**Supplementary Figure 4. Treg panel gating strategy (cont).** Helios, Ki67, CD15s, CD69 and FOXP3 expression was determined for CD4+CD25-FOXP3+, CD4+Teff, CD4+Treg and CD8+T lymphocytes. CD4+Treg subsets described by Miyara et al. (2009) were identified based on CD45RA and FOXP3 expression; FOXP3lowCD45RA+ resting Treg (rTreg), FOXP3loCD45RA- memory Treg (mTreg) and FOXP3hiCD45RA activated Treg (aTreg).

| 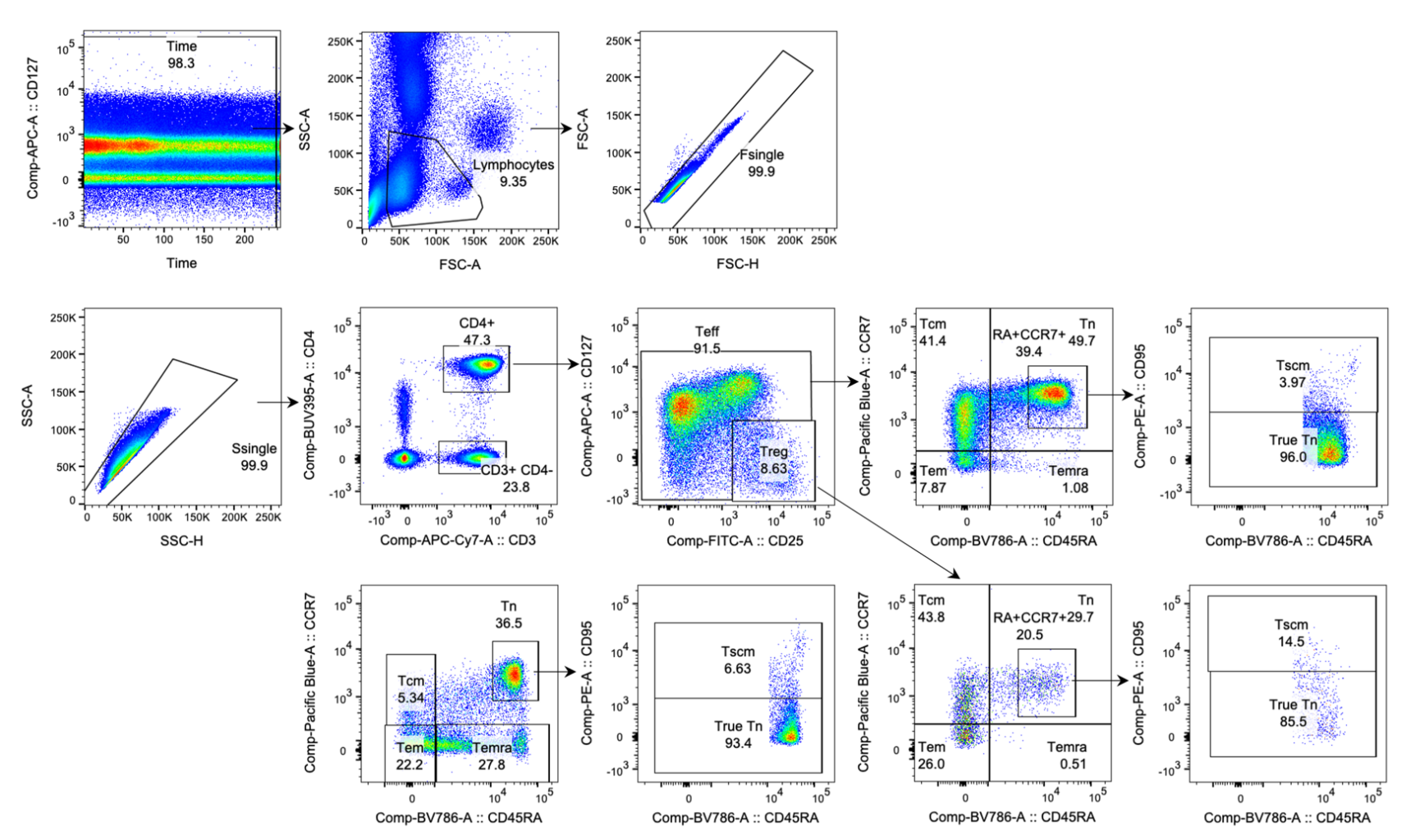 |
| --- |

**Supplementary Figure 5. T cell panel gating strategy.** All cells were first gated across time. Lymphocytes were gated based on FSC-area and SSC-area properties, followed by exclusion of doublets and debris by gating on FSC-height and FSC-area and by SSC-height and SSC-area. CD4+ and CD3+CD4-T lymphocytes were identified based on expression of CD3 and CD4. CD4+Teff and CD4+Tregs were determined based on expression of CD25 and CD127. The markers CD45RA and CCR7 were used to define the distinct differentiation subsets of CD4+Teff, CD4+Tregs and CD3+CD4-T lymphocytes; Tn, Tcm, Tem and Temra. Stringent gating of the CD45+CCR7+ naïve subset followed by CD95 expression allowed identification of the Tscm and True naïve subsets. Gating from parental populations is assigned with arrows.

| 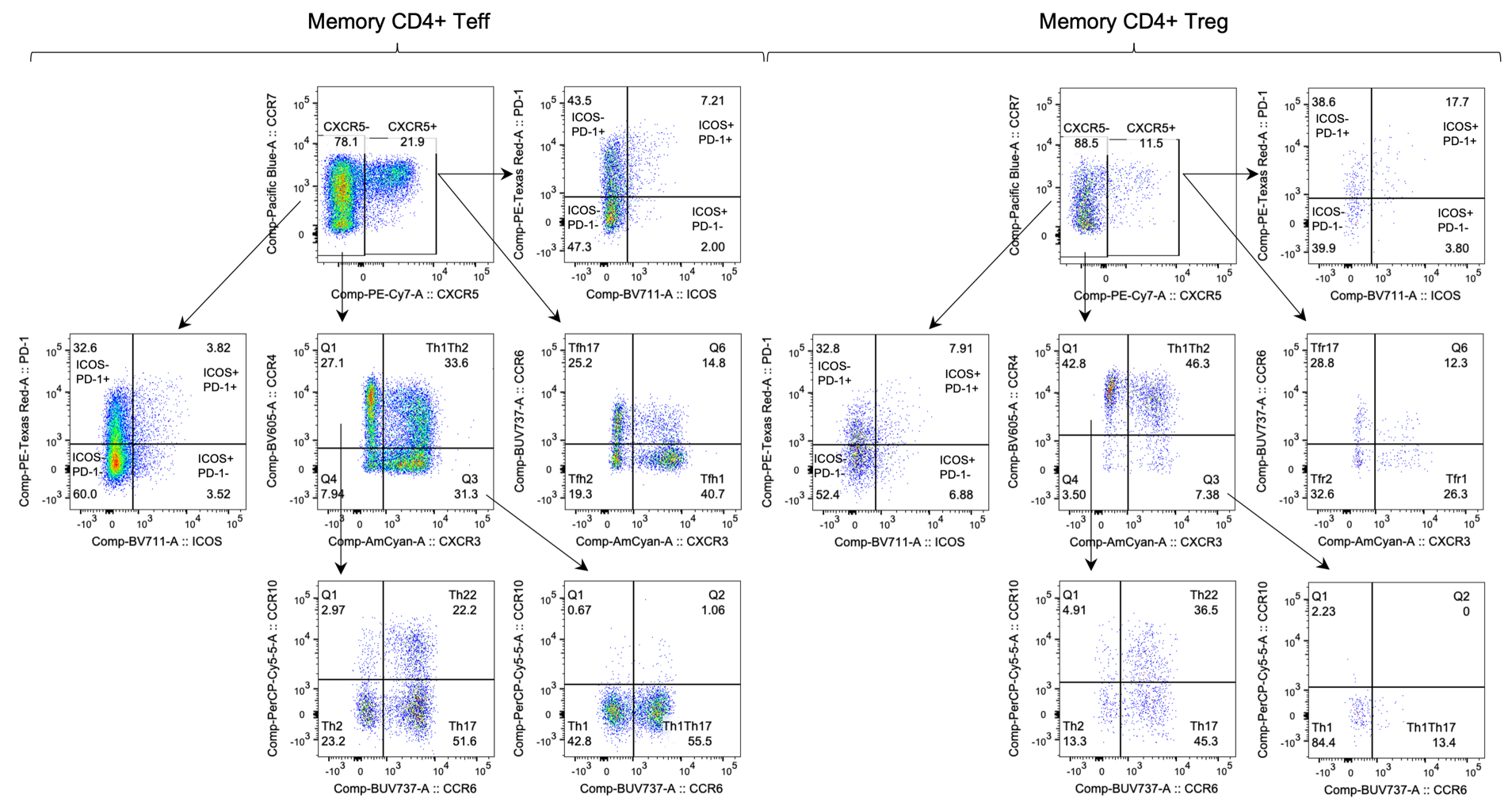 |
| --- |

**Supplementary Figure 5. T cell panel gating strategy (cont).**

Non-Tn cells were considered the total memory T cell population. CXCR5 expression allowed identification of T follicular helper (Tfh) cells (CXCR5+ memory CD4+Teff) and T follicular regulatory (Tfr) cells (CXCR5+ memory CD4+Treg) and PD-1 and ICOS expression were used to determine the different activation subsets. CXCR3 and CCR6 were used to define the Tfh1, Tfh2 and Tfh17 subsets and the Tfr1, Tfr2 and Tfr17 subsets. Expression of CXCR3, CCR4, CCR6 and CCR10 was used to identify the Th1, Th2, Th17 and Th1Th17 subsets of CXCR5- memory CD4+Teff and CXCR5- memory CD4+Tregs. PD-1 and ICOS expression identified the different activation subsets within the CD4+Teff and CD4+Treg memory populations. Gating from parental populations is assigned with arrows.

| **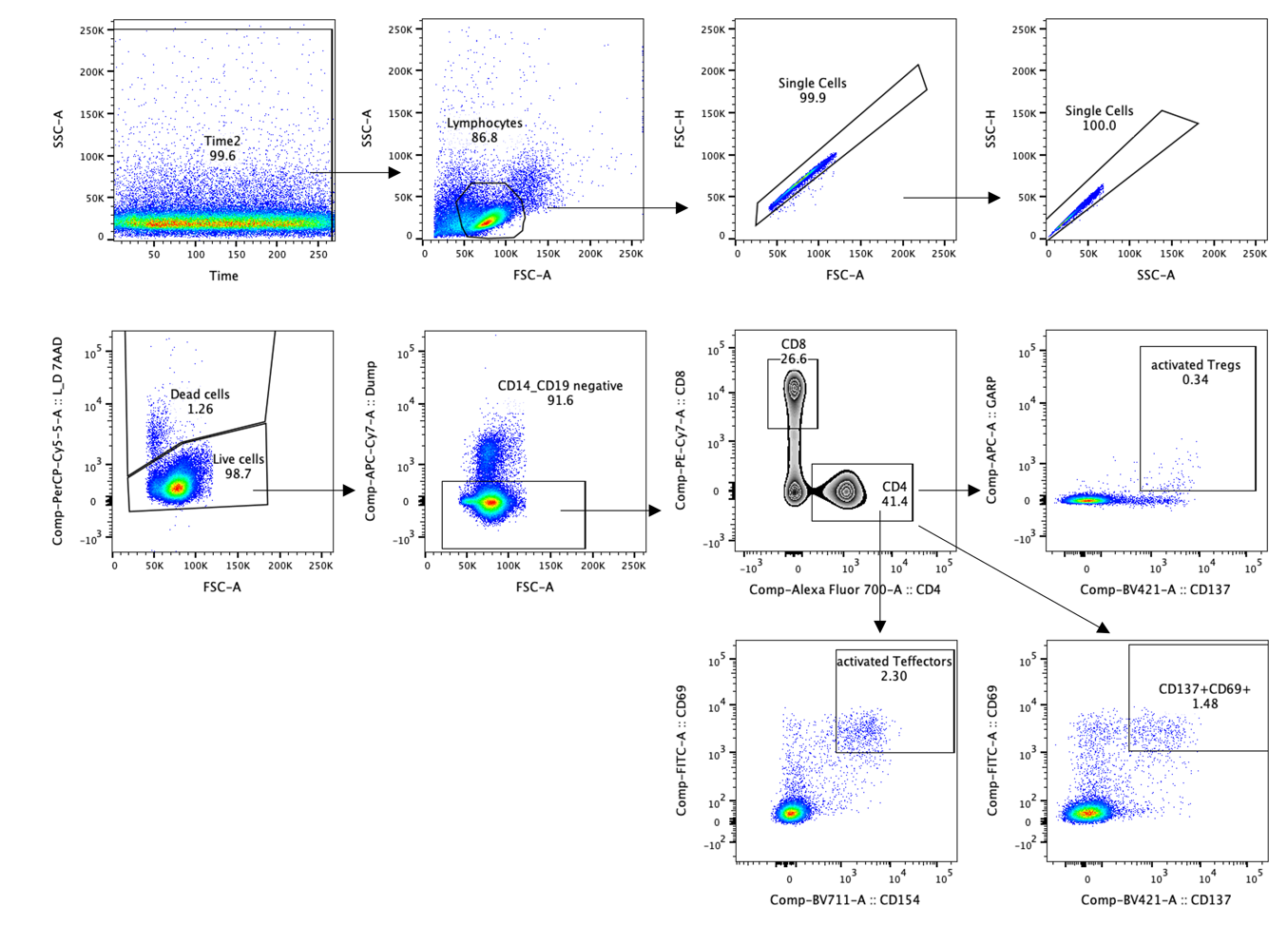** |
| --- |

**Supplementary Figure 6. Gating strategy for the identification of activated CD4+ T cells following stimulation with SEB.** All cells were first gated across time. Lymphocytes were gated based on FSC-area and SSC-area properties, followed by exclusion of doublets and debris by gating on FSC-height and FSC-area and by SSC-height and SSC-area. The L/D marker 7-AAD was used to identify live cells and CD14 and CD19 markers were used as DUMP channel to exclude CD14+ monocytes and CD19+ B lymphocytes. Expression of CD4 and CD8 was used to identify CD4+ T and CD8+ T lymphocytes. Three populations of SEB-specific CD4+ T cells were identified: Activated Teff (CD69+CD154+), activated CD4+ Tregs (GARP+CD137+) and CD137+CD69+ CD4+ T cells.

| 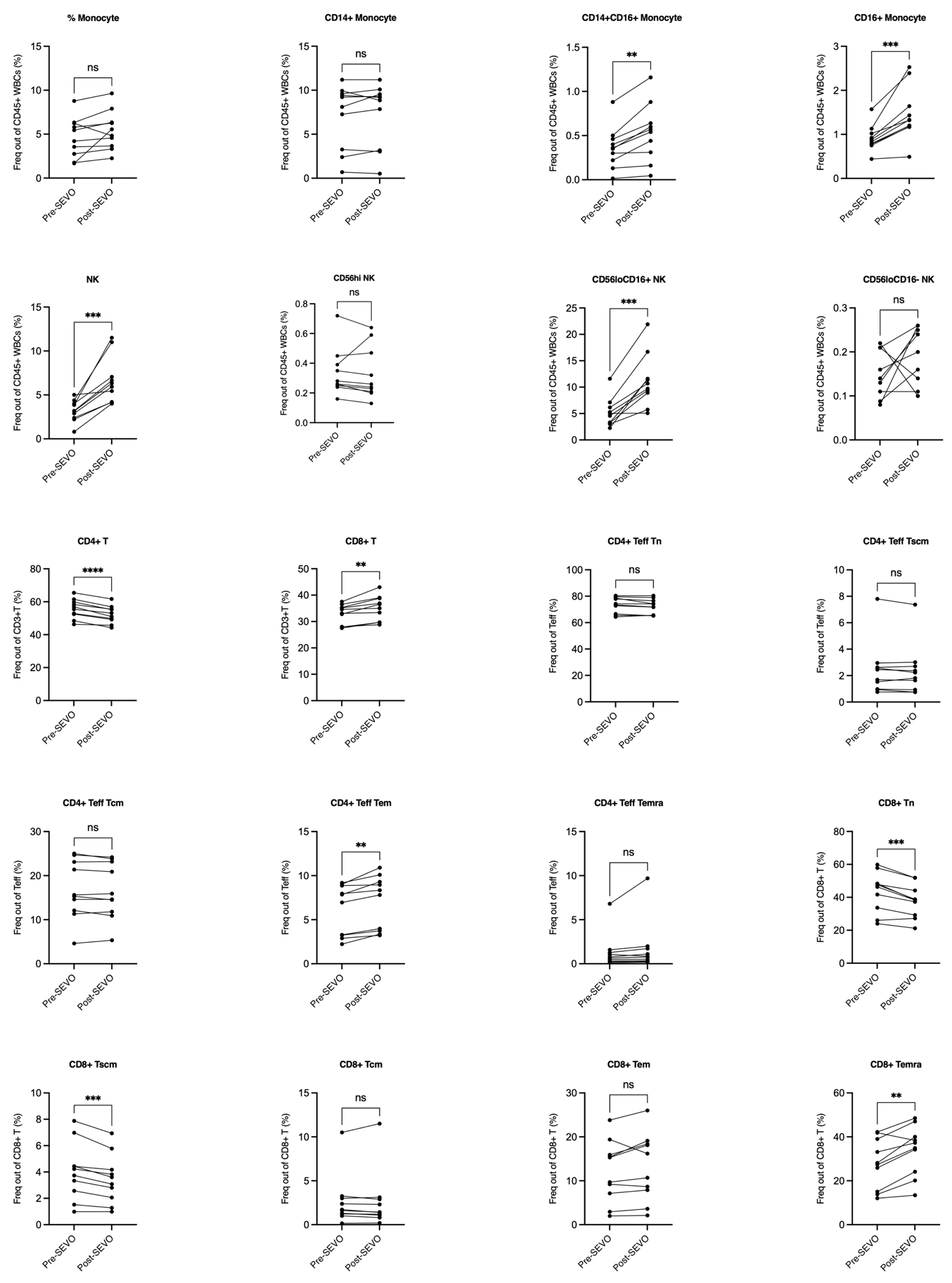 |
| --- |

| 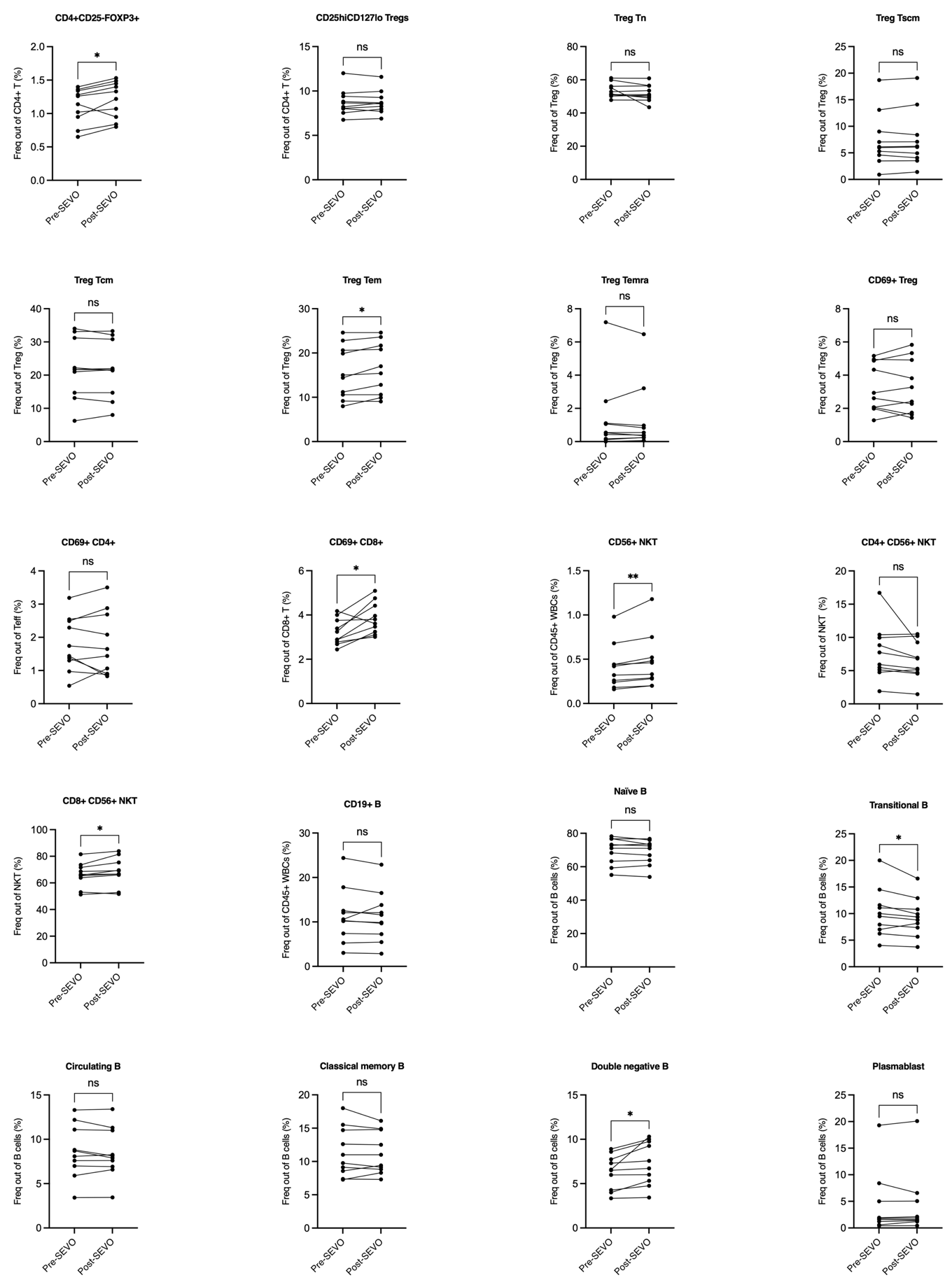 |
| --- |

**Supplementary Figure 7. Changes in cell frequency after inhaled anaesthetic induction in selected immune parameters.** Paired t tests performed without correction for multiple comparisons in selected immune cell subsets. Samples from the same individual are joined by a line. P values of <0.05*, <0.005**, <0.0005*** and <0.00005**** are shown.

| 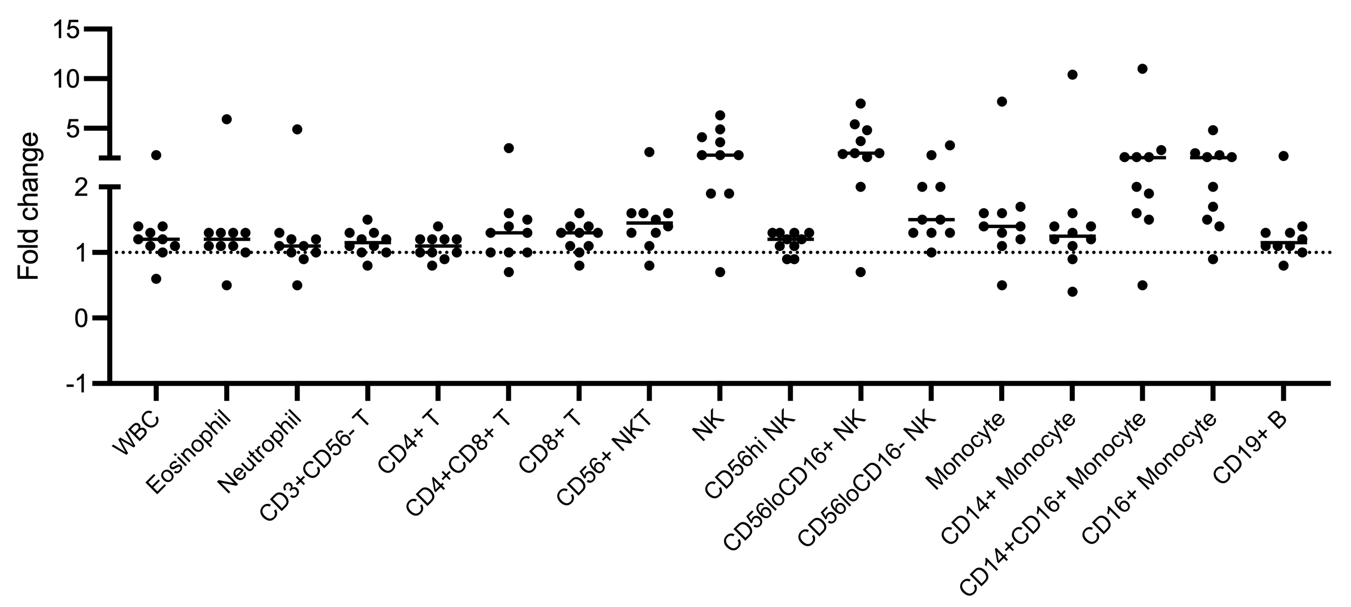 |
| --- |

**Supplementary Figure 8. Fold change in cell number after sevoflurane administration.** Fold change in cell number after inhaled anaesthetic induction. Mean fold change values are represented by a black horizontal line.

**References**

Miyara M, Yoshioka Y, Kitoh A, Shima T, Wing K, Niwa A, et al. Functional delineation and differentiation dynamics of human CD4+ T cells expressing the foxP3 transcription factor. Immunity. (2009) 30:899–911. doi: 10.1016/ J.IMMUNI.2009.03.019

| **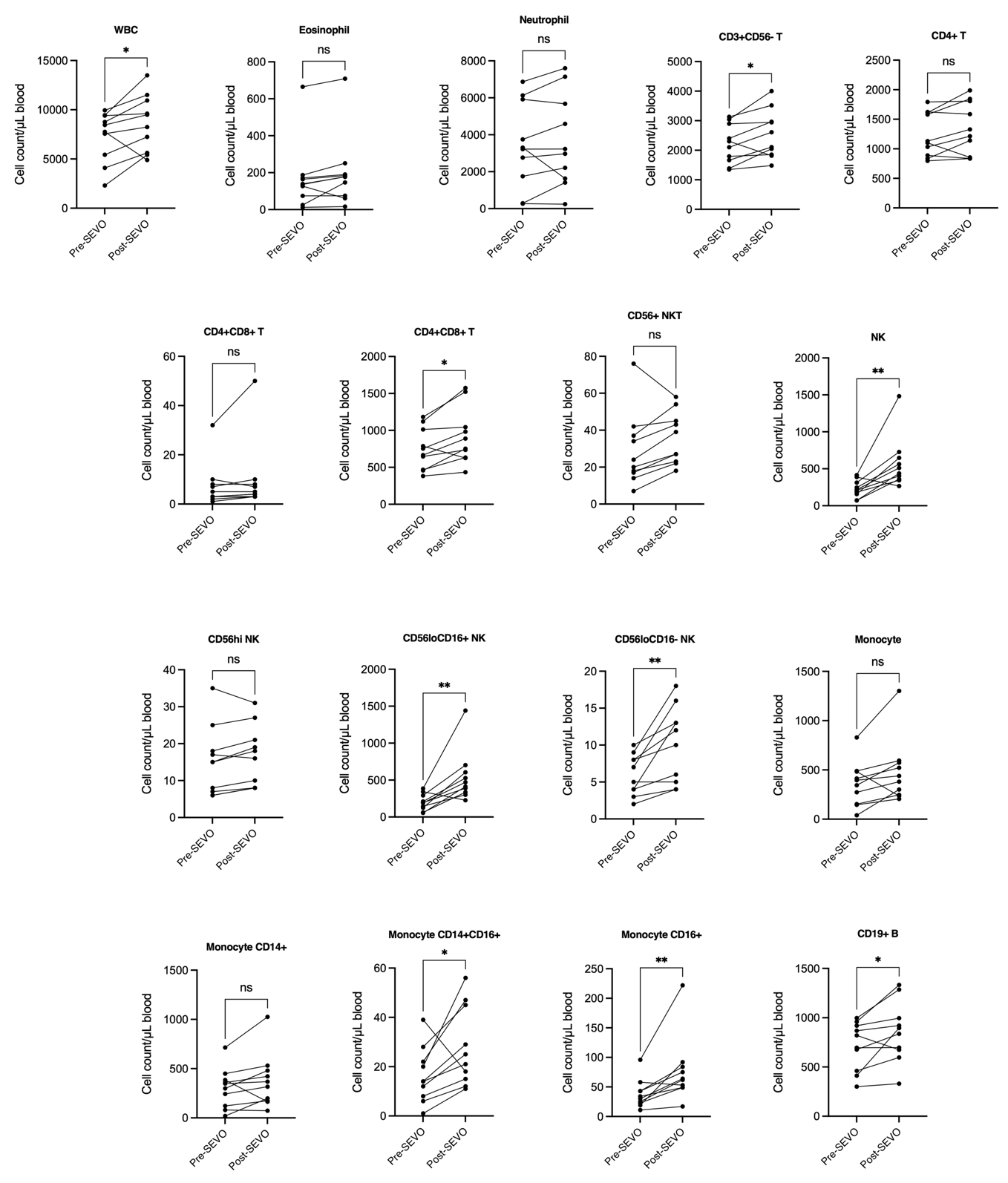** |
| --- |

**Supplementary Figure 9. Changes in cell number after sevoflurane administration in individual populations.** Paired t tests performed without correction for multiple comparisons in individual immune cell populations. Samples from the same individual are joined by a line. P values of <0.05*, <0.005**, <0.0005*** and <0.00005**** are shown.

| 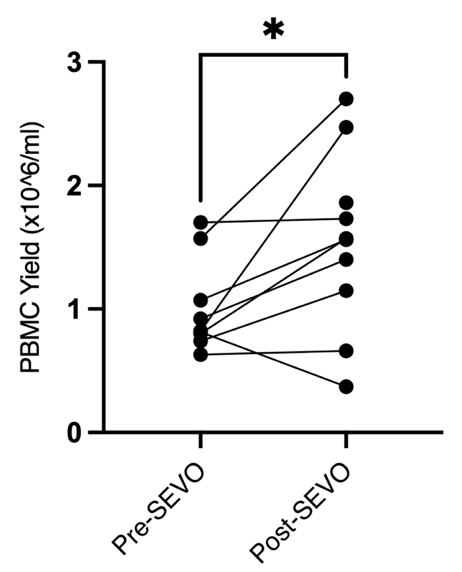 |
| --- |

**Supplementary Figure 10. PBMC yield before and after inhaled anaesthetic induction.** Paired t test performed on all samples showed a significant difference in PBMC yield before and after inhaled anaesthetic induction. Samples from the same individual are joined by a line. P value of <0.05* is shown.

**References**

Miyara, M., Yoshioka, Y., Kitoh, A., Shima, T., Wing, K., Niwa, A., Parizot, C., Taflin, C., Heike, T., Valeyre, D., Mathian, A., Nakahata, T., Yamaguchi, T., Nomura, T., Ono, M., Amoura, Z., Gorochov, G., & Sakaguchi, S. (2009). Functional Delineation and Differentiation Dynamics of Human CD4+ T Cells Expressing the FoxP3 Transcription Factor. *Immunity*, *30*(6), 899–911. <https://doi.org/10.1016/J.IMMUNI.2009.03.019>
